# A tensorial approach to the inversion of group-based phylogenetic models

**DOI:** 10.1186/s12862-014-0236-6

**Published:** 2014-12-04

**Authors:** Jeremy G Sumner, Peter D Jarvis, Barbara R Holland

**Affiliations:** School of Physical Sciences, University of Tasmania, Hobart TAS, 7001 Australia

**Keywords:** Groups, Representation theory, Symmetry, Markov chains

## Abstract

**Background:**

Hadamard conjugation is part of the standard mathematical armoury in the analysis of molecular phylogenetic methods. For group-based models, the approach provides a one-to-one correspondence between the so-called “edge length” and “sequence” spectrum on a phylogenetic tree. The Hadamard conjugation has been used in diverse phylogenetic applications not only for inference but also as an important conceptual tool for thinking about molecular data leading to generalizations beyond strictly tree-like evolutionary modelling.

**Results:**

For general group-based models of phylogenetic branching processes, we reformulate the problem of constructing a one-one correspondence between pattern probabilities and edge parameters. This takes a classic result previously shown through use of Fourier analysis and presents it in the language of tensors and group representation theory. This derivation makes it clear why the inversion is possible, because, under their usual definition, group-based models are defined for abelian groups only.

**Conclusion:**

We provide an inversion of group-based phylogenetic models that can implemented using matrix multiplication between rectangular matrices indexed by ordered-partitions of varying sizes. Our approach provides additional context for the construction of phylogenetic probability distributions on network structures, and highlights the potential limitations of restricting to group-based models in this setting.

## Background

Fundamental to evolutionary biology is the development and implementation of molecular phylogenetic methods [1]. These methods provide the means to reconstruct the past evolutionary history of biological entities given present-day molecular data, such as DNA. Considering Kimura’s neutral theory of molecular evolution, it is logical to apply a stochastic model at the level of DNA substitutions to construct probabilistic description of what molecular alignments are expected to be observed, given a proposed evolutionary history (tree topology and edge lengths). is commonly implemented assuming an IID (across sites in the alignment) and Markov process for DNA substitution, leading to a model that has a continuous-time Markov chain at its core (see Semple and Steel [2] for an introduction to the mathematics underlying modern phylogenetic methodology).

In a series of papers, Hendy and colleagues introduced the Hadamard conjugation as a novel tool for phylogenetic analyses [3–5]. They found an invertible relationship between a phylogenetic tree, as characterized by its edge length spectrum, and the probability distribution of site patterns (referred to as the sequence spectrum). Originally introduced only for the 2-state symmetric model, the Hadamard conjugation was later extended to the K3ST model [6–8] and further to any of the so-called “group-based” models [9]. Hadamard conjugation has been used as both a tool for simulation [10] and to look at statistical properties of methods, exploring the inconsistency of parsimony under a molecular clock [5,11]. For these sorts of applications, following the notation in Felsenstein [1], we can use the Hadamard transform *H* to start with an edge length spectrum *γ* and calculate the sequence spectrum *s*=*H*^−1^ log(*Hγ*). The beauty of Hadamard conjugations is that one can also begin with an observed sequence spectrum $\hat {s}$ and perform the inverse of the conjugation to empirically obtain an edge length spectrum $\hat {\gamma } = H^{-1}\log (H \hat {s})$. Although it is not expected that the $\hat {\gamma }$ spectrum will precisely match a tree, Hendy [12] proposed using an optimisation criterion to map from $\hat {\gamma }$ to the “closest tree”.

Several authors have commented that it is potentially a useful feature of Hadamard conjugation that data isn’t forced onto a fixed tree. The conflicting information can be retained and interpreted in the form of a “lentoplot” [13] or a splits-graph [14], with both of these methods implemented in *Spectronet* [15]. Schliep [16] gives some more statistical justification for such an approach by making a link to modern statistical techniques such as the Lasso and Ridge regression.

von Haeseler and Churchill [17] seems to be the first paper that explicitly suggests using Hadamard conjugation to provide a likelihood framework for networks. The principle idea in this work was to start with an edge length spectrum that encodes a set of incompatible splits, use the Hadamard transformation to get site probabilities and use these to determine a likelihood. This idea was further explored by Bryant [18], and Bryant [19] followed this through defining the “*n*-taxon process” for group-based models. It should be noted that likelihoods calculated via Hadamard are not equivalent to likelihoods calculated by taking a mixture of trees. Indeed, Matsen and Steel [20], Matsen et al. [21] used Hadamard methods in combination with phylogenetic invariants to show that mixtures of trees with the same topology can exactly mimic another tree under the 2-state model. Considering biological applications, thinking in terms of mixtures of trees or partitions where the data can be thought of as arising on a set of trees [22–24] seems more reasonable than the Hadamard conjugation. Strimmer and Moulton [25] suggested using split networks as a spring board to likelihood-based analyses on DAGs, but later identified several problems with the approach [26]; most notably, in split-networks internal nodes do not have a biological interpretation as an ancestor.

In Sumner et al. [27], we gave some additional insight into the interpretation of applying the Hadamard conjugation in a network setting. We showed that permutation group structure inherent to the Hadamard transformation – as for any group-based model – restricts the resulting process from being capable of reproducing truly convergent processes. This is a serious limitation, as one of the biological motivations for explicit network models is the ability to model convergent processes. We also presented an alternative algebraic formalism for the general Markov model, analogous to the *n*-taxon process, but capable of reproducing convergent processes.

From the point of view of group representation theory, the inversion of group-based models relies on the fact that the *irreducible* representations of an abelian group are one-dimensional, and the model structure essentially reduces to analysing group characters – hence the standard presentation of a Fourier inversion. In this article, we make this connection concrete. For the general Markov model, it is then immediately apparent that an analogous inversion is not possible because the algebraic structure underlying the model is not abelian and hence the irreducible representations are not one-dimensional. In fact, to obtain one-dimensional representations for the general Markov model, it is necessary to apply higher-degree polynomial maps (beyond the degree 1, linear case), and define “Markov invariants” [28]. These invariants present one-dimensional representations but at the cost of the higher degree – degree 5 in the case of the general Markov model with four states on quartet trees [29,30]. This connection between Hadamard transformation and Markov invariants is an interesting one, but we do not discuss it further here.

In this paper we approach the inversion of group-based phylogenetic models by taking a representation-theoretic perspective and working explicitly with tensor indices. Our approach rests heavily on the formalism of “phylogenetic tensors”, as presented in Bashford et al. [31], for the binary-symmetric and K3ST model, and Sumner et al. [27,28], for the general Markov model.

Although the main inversion results presented here are not more general than those in in Székely et al. [7], we think it is important to reformulate them using the language of tensors and representation theory. This viewpoint has already led to new approaches for modeling convergent evolution [27] and for studying non-group-based models [28]. However, in none of our previous work was the link to Hadamard conjugation explicitly discussed. By presenting an old technique (Hadamard conjugation) in a new light we hope to introduce other researchers to the viewpoint of tensor analysis and representation theory.

## Methods

### Group-based models

We consider the continuous-time formulation of Markov processes, and show how to implement the inversion of a group-based phylogenetic model based on *any* abelian group *G*. We note that such an inversion requires a map from tensor product space (where elements are indexed by ordered-*n*-partitions) to phylogenetic splits (where elements are indexed by bipartitions). We achieve this by finding canonical maps from bipartitions to ordered-*n*-partitions.

For a group *G* (not necessarily abelian) with order |*G*|=*d*, we write *G*={*σ*_1_,*σ*_2_,…,*σ*_*d*_}, and, when necessary, write *ε*∈*G* to specify the identity element of *G*. We will discuss the “regular representation” of *G* shortly, but skipping ahead we find that any rate matrix *Q* occurring in a group-based Markov model can be written in the form

(1) $$\begin{array}{@{}rcl@{}} \begin{aligned} Q=-\lambda\mathbf{1}+\sum_{\epsilon\neq \sigma\in G} \alpha^{\sigma}K_{\sigma}, \end{aligned} \end{array} $$

where each $0\leq \alpha ^{\sigma } \in \mathbb {R}$, $\lambda =\sum _{\epsilon \neq \sigma \in G}\alpha ^{\sigma }$ and the *K*_*σ*_ are the permutation matrices corresponding to the (non-identity) group elements *σ*∈*G*.

For the reader interested in deriving this result, consider the *d*-dimensional vector space $\left \langle G\right \rangle _{\mathbb {C}}\equiv \left \langle \sigma _{1},\sigma _{2},\ldots,\sigma _{d} \right \rangle _{\mathbb {C}}=\{v=v_{1}\sigma _{1}+v_{2}\sigma _{2}+\ldots +v_{d}\sigma _{d}:v_{i}\in \mathbb {C}\}$, with scalar multiplication and vector addition defined via

$$\begin{array}{@{}rcl@{}} \begin{aligned} v+\lambda v'&=(v_{1}\sigma_{1}+v_{2}\sigma_{2}+\ldots+v_{d}\sigma_{d})\\ &\quad\, +\lambda(v'_{1}\sigma_{1}+v'_{2}\sigma_{2}+\ldots+v'_{d}\sigma_{d})\\ & = (v_{1}+\lambda v'_{1})\sigma_{1}+(v_{2}+\lambda v'_{2})\sigma_{2}+\ldots \\ &\quad\, +(v_{d}+\lambda v'_{d})\sigma_{d}, \end{aligned} \end{array} $$

for all $v,v'\in \langle G\rangle _{\mathbb {C}}$ and $\lambda \in \mathbb {C}$. The *regular representation,*$\rho _{\text {reg}}: G \rightarrow GL(d,\mathbb {C})$, is then defined by setting the group action

$$\sigma :v\mapsto \sigma v=v_{1}(\sigma\sigma_{1})+v_{2}(\sigma\sigma_{2})+\ldots +v_{d}(\sigma\sigma_{d}), $$

for all $v\in \langle G\rangle _{\mathbb {C}}$ and *σ*∈*G*. If we fix {*σ*_1_,*σ*_2_,…,*σ*_*d*_} as an ordered basis for $\langle G \rangle _{\mathbb {C}}$, it is then clear – via Cayley’s theorem – that each group element *σ* gets mapped to a permutation matrix *K*_*σ*_:=*ρ*_reg_(*σ*), with $K_{\sigma }\sigma _{i}=\sum _{j}{\left [ K_{\sigma } \right ]}^{j}_{i}\sigma _{j}:=\sigma \sigma _{i}$. Thus *K*_*σ*_ has matrix elements

(2) $$\begin{array}{@{}rcl@{}} \begin{aligned} \left[K_{\sigma}\right]^{j}_{i}=\left\{ \begin{array}{l}1,~~~\text{if}\; \sigma_{j}=\sigma\sigma_{i}, \\ 0,~~~\text{otherwise.} \end{array}\right. \end{aligned} \end{array} $$

Consider the unit column vectors

$$\begin{aligned} \xi_{1}&=(1,0,0,\ldots,0)^{T},\quad \xi_{2}=(0,1,0,0,\ldots,0)^{T},\quad\ldots\quad \\ \xi_{d}&=(0,0,\ldots,0,1)^{T}; \end{aligned} $$

and identify each $\sigma _{i}\in \left \langle G\right \rangle _{\mathbb {C}}$ with $\xi _{i}\in \mathbb {C}^{d}$, so that the group action becomes *σ*:*ξ*_*i*_↦*K*_*σ*_*ξ*_*i*_=*ξ*_*j*_ where *σ*_*j*_=*σσ*_*i*_. Thus the matrix elements $\left [K_{\sigma }\right ]^{j}_{i}$ have *i* as the column label and *j* as the row label.

A group-based Markov model is then obtained by taking a continuous-time Markov chain with state space *G*={*σ*_1_,*σ*_2_,…,*σ*_*d*_} and using the group multiplication in *G* to assign a rate *α*_*σ*_ to all substitutions *σ*_1_↦*σ*_2_ where *σσ*_1_=*σ*_2_. Following this through (as is done in detail in [32]) we are led to the formula (1) for rate matrices in any group-based model.

The regular representation is one example of the general concept of a *representation* of *G* on a vector space *V*, defined as a homomorphism *ρ*:*G*→*GL*(*V*) satisfying *ρ*(*g*_1_*g*_2_)=*ρ*(*g*_1_)*ρ*(*g*_2_) for all *g*_1_,*g*_2_∈*G*. A representation is said to be *reducible* if there exists a proper subspace *U*⊂*V* satisfying *ρ*(*g*)*U*⊂*U*, i.e. the set of matrices *ρ*(*G*) send vectors in *U* back to *U*. In this case, *U* is called an *invariant subspace*. The representation *ρ* is then called *irreducible* if *V* does not contain any invariant subspaces.

The reader should note that the usual construction of a “group-based” model [2] stipulates that *G* be *abelian*. Although the construction just given using the regular representation allows for non-abelian *G*, we will nonetheless only consider the abelian case in this paper, because, as discussed in the introduction, it is only in the abelian case that a (linear) inversion of phylogenetic models is possible. In this case the irreducible representations of *G* are all one-dimensional [33], and hence the analysis reduces to computations with group characters, as is exploited in the previous approaches using Fourier analysis [9,34].

### Phylogenetic tensors

We denote [*d*]:={1,2,…,*d*} as the state space for a continuous-time Markov chain. Consider an *n*-taxa phylogenetic tree and a *d*-state phylogenetic pattern distribution $\{p_{i_{1}i_{2}\ldots i_{n}}\}_{i_{1},i_{2},\ldots,i_{n}\in {\left [ d \right ]}}$ with the interpretation that $p_{i_{1}i_{2}\ldots i_{n}}$ is the probability that the observed state at the *k*^*th*^ leaf on the tree is *i*_*k*_. As is shown in Sumner and Jarvis [35] and in more detail in Sumner et al. [27], such phylogenetic pattern distributions can be represented abstractly as tensors in the *n*-fold tensor product space $\otimes ^{n}\mathbb {C}^{d}:=\mathbb {C}^{d}\otimes \mathbb {C}^{d} \otimes \ldots \otimes \mathbb {C}^{d}$, as follows. If we choose {*ξ*_1_,*ξ*_2_,…,*ξ*_*d*_} as an ordered basis for $\mathbb {C}^{d}$, and ordered basis $\{\xi _{i_{1}}\otimes \xi _{i_{2}}\otimes \ldots \otimes \xi _{i_{d}}\}_{i_{1},i_{2},\ldots,i_{n}\in {\left [ d \right ]}}$ for the tensor product space, a “phylogenetic tensor” $P\in \otimes ^{n}\mathbb {C}^{d}$ is then defined as

$$\begin{array}{@{}rcl@{}} \begin{aligned}  P=\sum_{i_{1},i_{2},\ldots,i_{n} \in {\left[ d \right]}}p_{i_{1}i_{2}\ldots i_{n}}\xi_{i_{1}}\otimes \xi_{i_{2}}\otimes \ldots \otimes \xi_{i_{n}}. \end{aligned} \end{array} $$

For readers who are unfamiliar with tensor products, it is possible to understand the general concept via the definition of the “Kronecker” product of a *n*×*m* matrix *A* and a *n*^′^×*m*^′^ matrix *B* as the *nn*^′^×*mm*^′^ matrix given by



We can index the matrix *A*⊗*B* with row indicies *i*_1_*j*_1_=11,12,…,*nn*^′^ and column indices *j*_1_*j*_2_=11,12,…,*mm*^′^, i.e. generically $(A\otimes B)_{i_{1}j_{1},i_{2}j_{2}}=A_{i_{1}i_{2}}B_{j_{1}j_{2}}$ and specifically (*A*⊗*B*)_12,32_=*A*_13_*B*_22_. This point of view is useful if one wants to write out specific matrix representations of tensors, however, in the development that follows will focus heavily on the indexing of tensor components in the various cases discussed.

Suppose $\pi =\sum _{i\in {\left [ d \right ]}}\pi _{i}\xi _{i} \in \mathbb {C}^{d}$ represents the state distribution of a single taxa, i.e. *π*_*i*_ is the probability that a randomly chosen site in the sequence will be in state *i*. Now suppose a phylogenetic branching event occurs and the sequence is copied. The corresponding phylogenetic tensor $P=\sum _{i_{1},i_{2}\in {\left [ d \right ]}}p_{i_{1}i_{2}}\xi _{i_{1}}\otimes \xi _{i_{2}}$ representing the joint distribution of the two-taxa just after the branching event then has the property that $p_{i_{1}i_{2}}=\pi _{i_{1}}$ if *i*_2_=*i*_1_ and is zero otherwise. Thinking in terms of tensor operations, we find that phylogenetic branching events can be generated by a linear operator $\delta :\mathbb {C}^{d}\rightarrow \mathbb {C}^{d}\otimes \mathbb {C}^{d}$ determined by *δ*(*π*)=*P* and defined in general using our chosen basis as

$$\begin{array}{@{}rcl@{}} \begin{aligned} \delta(\xi_{i}):=\xi_{i}\otimes \xi_{i},\qquad \delta(\pi)&=\delta\left(\sum_{i} \pi_{i}\xi_{i}\right)=\sum_{i}\pi_{i}\delta(\xi_{i})\\ &=\sum_{i}\pi_{i}\xi_{i}\otimes \xi_{i}. \end{aligned} \end{array} $$

The remarkable fact for group-based models, central to the present article, is that the permutation matrices “intertwine” particularly simply with the branching operator:

$$\begin{array}{@{}rcl@{}} \begin{aligned} \delta(K_{\sigma} \xi_{i})=\delta(\xi_{\sigma(i)})=\xi_{\sigma(i)}\otimes \xi_{\sigma(i)}=K_{\sigma}\otimes K_{\sigma}\cdot \delta(\xi_{i}). \end{aligned} \end{array} $$

Thus, for any rate matrix *Q* arising from a group-based model, we have (via the linearity of *δ*):

(3) $$\begin{array}{@{}rcl@{}} \begin{aligned} \delta\cdot Q=\left(-\lambda \mathbf{1}\otimes \mathbf{1} + \sum_{\epsilon\neq\sigma\in G }\alpha^{\sigma} K_{\sigma}\otimes K_{\sigma}\right)\cdot \delta. \end{aligned} \end{array} $$

We also note that, since *Q* can be expressed a linear combination of permutation matrices representing elements in a group *G*, the matrix powers *Q*^2^,*Q*^3^,*Q*^4^… will also be expressible as linear combinations of the same permutation matrices (although precise expressions for the relevant coefficients may or may not be easily computable). Together with (3), this implies that, for any substitution matrix *e*^*Qt*^ arising from matrix exponentiation,

(4) $$\begin{array}{@{}rcl@{}} \begin{aligned} \delta \cdot e^{Qt}=e^{-\lambda}\exp \left({\sum_{\epsilon\neq\sigma\in G }\alpha^{\sigma} K_{\sigma}\otimes K_{\sigma}}\right)\cdot \delta. \end{aligned} \end{array} $$

**Figure 1:**
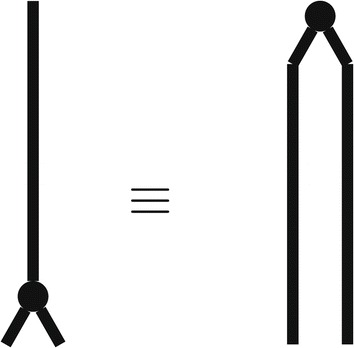
**Markov evolution on a single followed by a branching event (illustrated on the left), is equivalent to a branching event on a single taxon followed by correlated Markov evolution of two taxa (illustrated on the right).** Mathematically, this equivalence can be implemented by exploiting the equality given in (4).

This relation shows that mathematically, and hence conceptually, “Markov evolution on a single followed by a branching event” can be replaced with “Branching event on a single taxon followed by (correlated) Markov evolution of two taxa.” This equivalence is illustrated in Figure 1, and should be compared to the equivalent discussion of the “*n*-taxa process” given in [18] and [19].

In Sumner et al. [27] we showed how to generalise this intertwining action to the case of the general Markov model. Interestingly, for the general Markov model the appropriate intertwining has quite a different structure from what occurs in group-based models, and hence the simplicity of (4) is somewhat misleading in general. We refer the reader to Sumner et al. [27] for more discussion on this point.

Returning to the case of group-based models, for each subset *A*⊆[*n*], we define a linear map on $\otimes ^{n}\mathbb {C}^{d}$ as the tensor product $K^{(A)}_{\sigma }:=K_{\sigma }^{a_{1}}\otimes K_{\sigma }^{a_{2}} \otimes \ldots \otimes K_{\sigma }^{a_{n}}$ where *a*_*i*_=1 if *i*∈*A* and 0 otherwise. For example, if *n*=5, we have

$$\begin{array}{@{}rcl@{}} \begin{aligned} K_{\sigma}^{(\{1,2,4\})}=K_{\sigma} \otimes K_{\sigma} \otimes \mathbf{1} \otimes K_{\sigma} \otimes \mathbf{1}. \end{aligned} \end{array} $$

**Figure 2:**
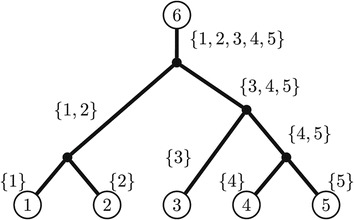
**A six taxa tree rooted at taxon 6 with edges labelled by subsets of {1,2,3,4,5}.**

To develop a phylogenetic tensor on a tree, we root the phylogenetic tree at taxon *n*, and label edges by subsets *∅*≠*e*⊆[*n*−1], where *i*∈*e* if the path from taxon *n* to taxon *i* crosses the edge labelled by *e*. A five taxon tree with this labelling, is presented in Figure 2. To each edge labelled by *∅*≠*e*⊆[*n*−1], we assign the rate matrix

$$\begin{array}{@{}rcl@{}} \begin{aligned} Q_{e}:=-\lambda_{e} \mathbf{1}+ \sum_{\epsilon\neq\sigma \in G}\alpha_{e}^{\sigma} K_{\sigma}, \end{aligned} \end{array} $$

where each $\alpha _{e}^{\sigma }\geq 0$ is the rate of substitution for all states *σ*_1_ to *σ*_2_ satisfying $\sigma =\sigma _{2}\sigma _{1}^{-1}$, and $\lambda _{e}=\sum _{\sigma \in G}\alpha _{e}^{\sigma }$. Each edge is then assigned substitution matrix $M_{e}=e^{Q_{e}}\vphantom {\dot {i}\!}$, so that the time parameter for each edge is absorbed into the definition of *Q*_*e*_.

Now iterating (4) multiple times, Bashford et al. [27,31] show that any phylogenetic tensor can be written as

(5) $$\begin{array}{@{}rcl@{}}\begin{aligned} P=e^{-\lambda}\exp\left(\sum_{\emptyset \neq e\subseteq {\left[ n-1 \right]},\sigma\in G} \alpha_{e}^{\sigma} K^{(e)}_{\sigma}\right)\cdot \delta^{n-1}\pi. \end{aligned} \end{array} $$

where $\lambda =\sum _{\emptyset \neq e\subseteq {\left [ n-1 \right ]}}\lambda _{e}=\sum _{\emptyset \neq e\subseteq {\left [ n-1 \right ]},\epsilon \neq \sigma \in G} \alpha _{e}^{\sigma }$, and *δ*^*n*−1^*π* is the *d*×*d*×…×*d* tensor that represents the “zero edge-length star tree” distribution on *n* taxa. It is this form of phylogenetic tensors that will do a lot of the heavy lifting in the discussion that follows. The reader should note that under this representation, there is no need for the edge parameters $\left \{\alpha _{e}^{\sigma }:\emptyset \neq e\subseteq {\left [ n-1 \right ]},\sigma \in G\right \}$ to be chosen to be compatible with a particular tree, hence the possibilities for generalising to non-tree-like or network models, as discussed in the introduction.

The stationary distribution for group-based models is uniform (because the rate matrices are doubly stochastic). In this paper we always assume a stationary distribution, so that:

$$\begin{array}{@{}rcl@{}} \begin{aligned} \pi=\textstyle{\frac{1}{d}}(1,1,\ldots,1)^{T}, \end{aligned} \end{array} $$

and *δ*^*n*−1^*π* has tensor components

$$\begin{array}{@{}rcl@{}} \begin{aligned} \left[\delta^{n-1}\pi\right]_{i_{1}i_{2}\ldots i_{n}}= \left\{ \begin{array}{ll} \frac{1}{d},&\;\;\text{if}\; i_{1}=i_{2}=\ldots=i_{n},\\ 0,&\;\;\text{otherwise.} \end{array}\right. \end{aligned} \end{array} $$

This concludes our discussion of the tensor presentation of phylogenetic probability distributions under group-based models. It is important to note that everything discussed so far works for any group-based model, with no requirement that the underlying group *G* be abelian.

In what follows, we discuss the inversion of *abelian* group-based models. We present the simplest case with $G=\mathbb {Z}_{2}$; the $G=\mathbb {Z}_{3}$ case; the $G=\mathbb {Z}_{2}\times \mathbb {Z}_{2}$ case; the general $G=\mathbb {Z}_{r}$ case; and finally we discuss the case of any abelian group.

## Results

### The binary-symmetric case

We begin with the inversion of the so-called “binary-symmetric” model. Consider $\mathbb {C}^{2}$ with standard basis

$$\begin{array}{@{}rcl@{}} \begin{aligned} \left\{\xi_{0}=\left(\begin{array}{r}1 \\ 0\end{array}\right),\xi_{1}=\left(\begin{array}{r}0 \\ 1\end{array}\right) \right\}. \end{aligned} \end{array} $$

As a group-based model, the binary-symmetric model arises by taking the group

$$\begin{array}{@{}rcl@{}} \begin{aligned} G:=\mathbb{Z}_{2}=\{0,1\}_{+ \text{(mod 2)}}\cong{\left\langle \sigma|\sigma^{2}=\epsilon \right\rangle}, \end{aligned} \end{array} $$

with a generic rate matrix given by

$$\begin{array}{@{}rcl@{}} \begin{aligned} Q=\left(\begin{array}{rr} -1 & 1 \\ 1 & -1 \\ \end{array}\right)=-\mathbf{1}+K, \end{aligned} \end{array} $$

where $K=\left (\begin {array}{rr} 0 & 1 \\ 1 & 0 \\ \end {array}\right)$ is the permutation matrix representing *σ* in the standard basis.

Now $\rho _{\text {reg}}:\mathbb {Z}_{2}\rightarrow \mathbb {M}_{2}(\mathbb {C})$, with *σ*↦*K*, is the regular representation of $\mathbb {Z}_{2}$, and the character table of $\mathbb {Z}_{2}$ given in Table 1 is easily recognised to be the Hadamard matrix

$$\begin{array}{@{}rcl@{}} \begin{aligned} h=\left(\begin{array}{rr} 1 & 1 \\ 1 & -1 \\ \end{array}\right). \end{aligned} \end{array} $$

**Table 1:** **The character table of** $\boldsymbol {\mathbb {Z}_{2}}$

	**id**	**sgn**
[ *e*]	1	1
[ *σ*]	1	-1

As $\mathbb {Z}_{2}$ is an abelian group, the irreducible representations are one-dimensional.

The corresponding projection operators can be read off from the columns of the character table. That is, the operators

$$\begin{array}{@{}rcl@{}} \begin{aligned} \Theta_{id}:=\textstyle{\frac{1}{2}}\left(\epsilon+\sigma\right),\qquad \Theta_{sgn}:=\textstyle{\frac{1}{2}}\left(\epsilon-\sigma\right); \end{aligned} \end{array} $$

project *ρ*_reg_=*id*⊕*sgn* onto the *id* and *sgn* representations of $\mathbb {Z}_{2}$, respectively.

This observation prompts us to work in the alternative basis:

$$\begin{array}{@{}rcl@{}} \begin{aligned} f_{0}:&=\Theta_{id}\cdot \xi_{0}=\Theta_{id}\cdot \xi_{1}=h\xi_{0}=\xi_{0}+\xi_{1},\\ f_{1}:&=\Theta_{sgn}\cdot \xi_{0}=-\Theta_{sgn}\cdot \xi_{1}=h\xi_{1}=\xi_{0}-\xi_{1}. \end{aligned} \end{array} $$

In this basis the permutation matrix is diagonal:

$$\begin{array}{@{}rcl@{}} \begin{aligned} \widehat{K}:=hKh^{-1}=\left(\begin{array}{rr} 1 & 0 \\ 0 & -1 \\ \end{array}\right),\qquad\!\!\! \widehat{Q}:=-\mathbf{1}+\widehat{K}=\left(\begin{array}{rr} 0 & 0 \\ 0 & -2 \\ \end{array}\right). \end{aligned} \end{array} $$

The representation-theoretic perspective on $\widehat {K}$ is to observe that *id*(*σ*)=1 and *sgn*(*σ*)=−1.

Referring to (5), we know that we can write a generic phylogenetic tensor as

$$\begin{array}{@{}rcl@{}} \begin{aligned} P=e^{-\lambda}\exp\left(\sum_{\emptyset \neq e\subseteq{\left[ n-1 \right]}}\alpha_{e}K^{(e)}\right)\cdot \delta^{n-1}\pi, \end{aligned} \end{array} $$

where $\lambda =\sum _{\emptyset \neq e\subseteq {\left [ n-1 \right ]}}\alpha _{e}$.

We index matrix and tensor indices by using $i,j,k=0,1\in \mathbb {Z}_{2}$ and allow multiplication × in the ring of integers . The Hadamard matrix then has matrix elements ${[\!h]^{j}_{i}}=(-1)^{i\times j}$ where *j* is the row index and *i* is the column index. Observe that in the diagonal basis, the permutation matrix has elements

$$\begin{array}{@{}rcl@{}} \begin{aligned} \left[\widehat{K}\right]_{i}^{j}=\delta_{ij}(-1)^{i}. \end{aligned} \end{array} $$

Thus we have expressions such as

$$\begin{array}{@{}rcl@{}} \begin{aligned} {\left[ \widehat{K}^{(\{2,3\})} \right]}_{i_{1}i_{2}i_{3}}^{j_{1}j_{2}j_{3}}=\delta_{i_{1}j_{1}} \delta_{i_{2}j_{2}}\delta_{i_{3}j_{3}}(-1)^{i_{2}+i_{3}}, \end{aligned} \end{array} $$

where $\widehat {K}^{(\{2,3\})}=\mathbf {1} \otimes \widehat {K}\otimes \widehat {K}$.

As we are dealing with tensors of arbitrary size, it is convenient to represent a string such as *i*_1_*i*_2_…*i*_*n*_ as an *ordered-bipartitionμ*=*μ*_0_*:μ*_1_ of the set [*n*], where *μ*_0_,*μ*_1_⊆[*n*] with *j*∈*μ*_*k*_ if and only if *i*_*j*_=*k*. For example we have the following equivalences:

$$\begin{array}{@{}rcl@{}} \begin{aligned} 00110& \equiv \{1,2,5\}\mathord{:}\{3,4\},\quad 01111\equiv \{1\}\mathord{:}\{2,3,4,5\},\quad \\ 10001& \equiv \{2,3,4\}\mathord{:}\{1,5\} \end{aligned} \end{array} $$

and inequivalence:

$$\begin{array}{@{}rcl@{}} \begin{aligned} 01010\equiv \{1,3,5\}\mathord{:}\{2,4\}\neq \{2,4\}\mathord{:}\{1,3,5\}\equiv 10101. \end{aligned} \end{array} $$

We then have

$$\begin{array}{@{}rcl@{}} \begin{aligned} {\left[ \widehat{K}^{(e)} \right]}_{i_{1}i_{2}\ldots i_{n}}^{j_{1}j_{2}\ldots j_{n}}& ={\left[ \widehat{K}^{(e)} \right]}_{\mu}^{\nu}={\left[ \widehat{K}^{(e)} \right]}_{\mu_{0}\mathord{:}\mu_{1}}^{\nu_{0}\mathord{:}\nu_{1}}\\ &=\delta_{\mu_{0}\nu_{0}}\delta_{\mu_{1}\nu_{1}}(-1)^{|e\cap \mu_{1}|}. \end{aligned} \end{array} $$

Defining *h*^(*n*)^:=*h*^(*n*−1)^⊗*h* where *h*^(1)^:=*h*, in the diagonal basis $\widehat {P}:=h^{(n)}\cdot P$ and using our notation *h*^(*n*)^ has tensor components

$$\begin{array}{@{}rcl@{}} \begin{aligned} {\left[ h^{(n)} \right]}_{\mu}^{\nu}={\left[h^{(n)}\right]}_{\mu_{0}\mathord{:}\mu_{1}}^{\nu_{0}\mathord{:}\nu_{1}}&={\left[ h^{(n)} \right]}_{i_{1}i_{2}\ldots i_{n}}^{j_{1}j_{2}\ldots j_{n}}\\ &=(-1)^{i_{1}\times j_{1}+i_{2}\times j_{2}+\ldots +i_{n}\times j_{n}}\\ &=(-1)^{|\mu_{1}\cap \nu_{1}|}. \end{aligned} \end{array} $$

The zero edge-length star-tree initial distribution has tensor components

$$\begin{array}{@{}rcl@{}} \begin{aligned} {\left[ \delta^{n-1}\pi \right]}_{i_{1}i_{2}\ldots i_{n}}=\textstyle{\frac{1}{2}}\delta_{i_{1}i_{2}}\delta_{i_{1}i_{3}}\ldots \delta_{i_{1}i_{n}}, \end{aligned} \end{array} $$

(where, although it seems we have given preference to taxon 1 in this expression, there are many ways that this distribution can be expressed using the *δ*_*ij*_). In the diagonal basis with $\widehat {\delta ^{n-1}\pi }:=h^{(n)}\cdot \delta ^{n-1}\pi $, we have components

$$\begin{array}{@{}rcl@{}} \begin{aligned} &{\left[ \widehat{\delta^{n-1}\pi} \right]}_{i_{1}i_{2}\ldots i_{n}}\\ &\quad=\textstyle{\frac{1}{2}}\sum_{j_{1},j_{2},\ldots,j_{n}}(-1)^{i_{1}\times j_{1}+i_{2}\times j_{2}+\ldots +i_{n}\times j_{n}}\delta_{j_{1}j_{2}}\delta_{j_{1}j_{3}}\ldots \delta_{j_{1}j_{n}}\\ &\quad=\textstyle{\frac{1}{2}}\sum_{j_{1}}(-1)^{\left(i_{1}+i_{2}+\ldots +i_{n}\right)\times j_{1}} =\textstyle{\frac{1}{2}}\left(1\,+\,(-1)^{i_{1}+i_{2}+\ldots +i_{n}}\right), \end{aligned} \end{array} $$

which is exactly the statement

$$\begin{array}{@{}rcl@{}} \begin{aligned} {\left[\widehat{\delta^{n-1}\pi} \right]}_{\mu}={\left[\widehat{\delta^{n-1}\pi} \right]}_{\mu_{0}\mathord{:}\mu_{1}}=\textstyle{\frac{1}{2}}\left(1+(-1)^{|\mu_{1}|}\right). \end{aligned} \end{array} $$

Since $\widehat {K}$ is diagonal in the transformed basis, we can conclude that

$$\begin{array}{@{}rcl@{}} \begin{aligned} {\left[\widehat{P} \right]}_{\mu}&={\left[\widehat{P}\right]}_{\mu_{0}\mathord{:}\mu_{1}}\\ &=e^{-\lambda}\exp\!\left(\sum_{\emptyset \neq e\subseteq {\left[ 2,n \right]}}\!\alpha_{e}{\left[ \widehat{K}^{(e)} \right]}^{\mu_{0}\mathord{:}\mu_{1}}_{\mu_{0}\mathord{:}\mu_{1}}\!\right)\textstyle{\frac{1}{2}}\left(1\,+\,(-1)^{|\mu_{1}|}\right). \end{aligned} \end{array} $$

Of course many of these tensor components will be zero and we would like to ignore these.

Take *u*=*u*_0_*:u*_1_ as an ordered bipartition of the reduced set [*n*−1], so that *u*≡*i*_1_*i*_2_…*i*_*n*−1_ where *j*∈*u*_*k*_ if and only if *i*_*j*_=*k*, and define

$$\begin{array}{@{}rcl@{}} \begin{aligned} \gamma(u)&=\left\{\begin{array}{ll} 0,~\text{if }|u_{1}|~\text{is even,}\\1,~\text{if }|u_{1}|~\text{is odd;}\end{array}\right.\\ &=2-\left(0|u_{0}|+1|u_{1}|\right)\text{(mod 2)}, \end{aligned} \end{array} $$

and interpret *u*·*γ*(*u*) as a string: *u*·*γ*(*u*) = *i*_1_*i*_2_…*i*_*n*−1_*γ*(*u*).

If we make the definitions

$$\begin{array}{@{}rcl@{}}\begin{aligned} \mathcal{P}_{u}:={\left[\widehat{P} \right]}_{u\cdot \gamma(u)},\qquad \eta_{u}:=\textstyle{\frac{1}{2}}\sum_{\emptyset \neq e\subseteq {\left[ n-1 \right]}}\alpha_{e}{\left[ \widehat{K}^{(e)} \right]}^{u\cdot \gamma(u)}_{u\cdot \gamma(u)}, \end{aligned} \end{array} $$

then we can write the non-zero components as

$$\begin{array}{@{}rcl@{}} \begin{aligned} \mathcal{P}_{u}=e^{-\lambda}\exp\left(\eta_{u}\right), \end{aligned} \end{array} $$

with inverses

(6) $$\begin{array}{@{}rcl@{}} \begin{aligned} \eta_{u}=\ln\left(\mathcal{P}_{u}\right)+\lambda. \end{aligned} \end{array} $$

This is the first part of the inversion.

We would like to go further and actually recover the individual edge weights *α*_*e*_. To do this we define the (square) 2^*n*−1^×2^*n*−1^ matrix *F* with components

$$\begin{array}{@{}rcl@{}} \begin{aligned} {\left[ F \right]}_{u}^{e}:={\left[ \widehat{K}^{(e)} \right]}^{u\cdot \gamma(u)}_{u\cdot \gamma(u)}=(-1)^{|e\cap u|}={\left[ h^{(n-1)} \right]}^{e}_{u}, \end{aligned} \end{array} $$

with *e* a subset and *u* an ordered-bipartition of [*n*−1]. As $\left (h^{(n-1)}\right)^{2}=\textstyle {\frac {1}{2^{n-1}}}\mathbf {1}$, we see that *F* provides its own inverse *F*^−1^ with components

$$\begin{array}{@{}rcl@{}} \begin{aligned} {\left[ F^{-1} \right]}_{e}^{u}:=\textstyle{\frac{1}{2^{n-1}}}{\left[ F \right]}_{u}^{e}. \end{aligned} \end{array} $$

Defining the column vectors $\vec {\alpha }=\left \{\alpha _{e}\right \}$ and $\vec {\eta }=\left \{\eta _{u}\right \}$, we can write the matrix equations

$$\begin{array}{@{}rcl@{}} \begin{aligned} \vec{\eta}=F\vec{\alpha},\qquad \vec{\alpha}=F^{-1}\vec{\eta}. \end{aligned} \end{array} $$

Together with the first part of the inversion (6), these equations give a one-one map between pattern probabilities and edge weights for the binary-symmetric model.

### Inversion of the $\mathbb {Z}_{3}$ model

Taking confidence from the previous case we now discuss the inversion of the group-based phylogenetic model with $G=\mathbb {Z}_{3}$. We take

$$\mathbb{Z}_{3}=\{0,1,2\}_{+\text{(mod 3)}}\cong{\langle \sigma|\sigma^{3}=\epsilon \rangle}, $$

and, by analogy to the $\mathbb {Z}_{2}$ case, index tensors with indices *i*,*j*=0,1,2 and allow multiplication × by extending $\mathbb {Z}_{3}$ to the ring $\mathbb {F}_{3}=\{0,1,2\}_{+,\times \text {(mod 3)}}$.

In this case a generic rate matrix is given by

$$\begin{array}{@{}rcl@{}} \begin{aligned} Q&=\left(\begin{array}{ccc} -(\alpha+\beta) & \beta & \alpha \\ \alpha & -(\alpha+\beta) & \beta \\ \beta & \alpha & -(\alpha+\beta) \\ \end{array} \right)\\ &=\,-\left(\alpha+\beta\right)\mathbf{1}+\alpha K_{1}+\beta K_{2}, \end{aligned} \end{array} $$

where

$$\begin{array}{@{}rcl@{}} \begin{aligned} K_{1}=\left(\begin{array}{ccc} 0 & 0 & 1 \\ 1 & 0 & 0 \\ 0 & 1 & 0 \\ \end{array}\right),\qquad K_{2}=\left(\begin{array}{ccc} 0 & 1 & 0 \\ 0 & 0 & 1 \\ 1 & 0 & 0 \\ \end{array}\right), \end{aligned} \end{array} $$

are the matrices representing the permutations *σ*≅(123) and *σ*^2^≅(132) under the regular representation, respectively.

We define *ω*=*e*^2*πi*/3^, and present the character table of $\mathbb {Z}_{3}$ in Table 2. The decomposition of the regular representation is *ρ*_reg_=*id*⊕*ω*⊕*ω*^2^, and the columns of the character table give the projection operators onto the (one-dimensional) irreducible subspaces:

$$\begin{array}{@{}rcl@{}} \begin{aligned} \Theta_{id}:&=\textstyle{\frac{1}{3}}\left(\epsilon+\sigma+\sigma^{2}\right),\\ \Theta_{\omega}:&=\textstyle{\frac{1}{3}}\left(\epsilon+\omega\sigma+\omega^{2}\sigma^{2}\right),\\ \Theta_{\omega^{2}}:&=\textstyle{\frac{1}{3}}\left(\epsilon+\omega^{2}\sigma+\omega\sigma^{2}\right). \end{aligned} \end{array} $$

**Table 2:** **The character table of** $\boldsymbol {\mathbb {Z}_{3}}$

	**id**	***ω***	***ω*** ^**2**^
[ *e*]	1	1	1
[ *σ*]	1	*ω*	*ω* ^2^
[ *σ* ^2^]	1	*ω* ^2^	*ω*

Therefore, the matrix

$$\begin{array}{@{}rcl@{}} \begin{aligned} f=\left(\begin{array}{ccc} 1 & 1 & 1 \\ 1 & \omega & \omega^{2} \\ 1 & \omega^{2} & \omega \\ \end{array}\right), \end{aligned} \end{array} $$

diagonalizes the generic rate matrix for this model:

$$\begin{array}{@{}rcl@{}} \begin{aligned} \widehat{Q}=fQf^{-1}= \left(\begin{array}{ccc} 0 & 0 & 0 \\ 0 & \alpha\omega+\beta\omega^{2} & 0 \\ 0 & 0 & \alpha\omega^{2}+\beta\omega \\ \end{array}\right), \end{aligned} \end{array} $$

or, equivalently,

$$\begin{array}{@{}rcl@{}} \begin{aligned} \widehat{K}_{1}&={fK}_{1} f^{-1}= \left(\begin{array}{ccc} 1 & 0 & 0 \\ 0 & \omega & 0 \\ 0 & 0 & \omega^{2} \\ \end{array}\right),\qquad\\ \widehat{K}_{2}&={fK}_{2} f^{-1}= \left(\begin{array}{ccc} 1 & 0 & 0 \\ 0 & \omega^{2} & 0 \\ 0 & 0 & \omega \\ \end{array}\right). \end{aligned} \end{array} $$

We recall our basic result (5) that for group-based models, a generic phylogenetic tensor can be expressed as

$$\begin{array}{@{}rcl@{}} \begin{aligned} P=e^{-\lambda}\exp\left(\sum_{\emptyset \neq e\subseteq{\left[ n-1 \right]}} \left(\alpha_{e}K^{(e)}_{1}+\beta_{e}K^{(e)}_{2}\right)\right)\cdot \delta^{n-1}\pi, \end{aligned} \end{array} $$

where $\lambda =\sum _{\emptyset \neq e\subseteq {\left [ n-1 \right ]}}\left (\alpha _{e}+\beta _{e}\right)$. We take the stationary distribution as initial distribution, so $\pi =\left (\textstyle {\frac {1}{3}},\textstyle {\frac {1}{3}},\textstyle {\frac {1}{3}}\right)^{T}$.

The matrix elements of *f* can be expressed as ${[f]_{i}^{j}}=\omega ^{i\times j}$, where we extend $i,j\in \mathbb {Z}_{3}$ to include multiplication × from the ring of integers . Similarly,

$$\begin{array}{@{}rcl@{}} \begin{aligned} {\left[ \widehat{K}_{1} \right]}_{i}^{j}=\delta_{ij}\omega^{i},\qquad {\left[ \widehat{K}_{2} \right]}_{i}^{j}=\delta_{ij}(\omega^{2})^{i}. \end{aligned} \end{array} $$

More generally, tensorial components can be expressed as

$$\begin{array}{@{}rcl@{}} \begin{aligned} {\left[ \mathbf{1}\otimes \widehat{K_{1}}\otimes \widehat{K_{1}} \right]}_{i_{1}i_{2}i_{3}}^{j_{1}j_{2}j_{3}}=\delta_{i_{1}j_{1}}\delta_{i_{2}j_{2}} \delta_{i_{3}j_{3}}\omega^{i_{2}+i_{3}}. \end{aligned} \end{array} $$

We represent a string *i*_1_*i*_2_…*i*_*n*_ as an *ordered-tripartition*, *i*_1_*i*_2_…*i*_*n*_≡*μ*=*μ*_0_*:μ*_1_*:μ*_2_, of the set [*n*], where *j*∈*μ*_*k*_ if and only if *i*_*j*_=*k*. For example, if we take *n*=5, we have:

$$\begin{array}{@{}rcl@{}}\begin{aligned} 00000&\equiv \{1,2,3,4,5\}\mathord{:}\emptyset \mathord{:}\emptyset, \quad 20120\equiv \{2,5\}\mathord{:} \{3\}\mathord{:} \{1,4\},\\ 01122&\equiv \{1\}\mathord{:} \{2,3\}\mathord{:} \{4,5\}. \end{aligned} \end{array} $$

Taking *n* = 3, we have

$$\begin{array}{@{}rcl@{}} \begin{aligned} {\left[ \widehat{K}_{1}^{(\{2,3\})} \right]}_{\mu}^{\nu}= {\left[ \mathbf{1}\otimes \widehat{K}_{1}\otimes \widehat{K}_{1} \right]}_{\mu}^{\nu}&={\left[ \mathbf{1}\otimes \widehat{K}_{1}\otimes \widehat{K}_{1} \right]}_{\mu_{0}\mathord{:} \mu_{1}\mathord{:} \mu_{2}}^{\nu_{0}\mathord{:} \nu_{1}\mathord{:} \nu_{2}}\\ &=\delta_{\mu\nu}\omega^{|\mu_{1}\cap \{2,3\}|+2|\mu_{2}\cap \{2,3\}|}, \end{aligned} \end{array} $$

and in general:

$$\begin{array}{@{}rcl@{}} \begin{aligned} {\left[ \widehat{K}^{(e)}_{1} \right]}_{\mu}^{\nu}&=\delta_{\mu\nu}\omega^{|e\cap \mu_{1}|+2|e\cap \mu_{2}|},\qquad \\ {\left[ \widehat{K}^{(e)}_{2} \right]}_{\mu}^{\nu}&=\delta_{\mu\nu}\omega^{|e\cap \mu_{2}|+2|e\cap \mu_{1}|}. \end{aligned} \end{array} $$

Taking the uniform distribution as initial distribution, the initial star-tree distribution can be written as

$$\begin{array}{@{}rcl@{}} \begin{aligned} {\left[\delta^{n-1}\pi \right]}_{i_{1}i_{2}\ldots i_{n}}=\textstyle{\frac{1}{3}}\delta_{i_{1}i_{2}}\delta_{i_{1}i_{3}}\ldots \delta_{i_{1}i_{n}}. \end{aligned} \end{array} $$

Defining *f*^(*n*)^=*f*^(*n*−1)^⊗*f* where *f*^(1)^=*f*, we have

$$\begin{array}{@{}rcl@{}} \begin{aligned} {\left[\, f^{(n)} \right]}_{\mu}^{\nu}&={\left[ f^{(n)} \right]}_{i_{1}i_{2}\ldots i_{n}}^{j_{1}j_{2}\ldots j_{n}}={\left[\, f \right]}_{i_{1}}^{j_{1}}{\left[\, f \right]}_{i_{2}}^{j_{2}}\ldots{\left[\, f \right]}_{i_{n}}^{j_{n}} \\ &=\omega^{i_{1}\times j_{1}+i_{2}\times j_{2}+\ldots +i_{n}\times j_{n}}, \end{aligned} \end{array} $$

and in the transformed basis, where $\widehat {\delta ^{n-1}\pi }:=f^{(n)}\cdot \delta ^{n-1}\pi $, we have

$$\begin{array}{@{}rcl@{}} \begin{aligned} {\left[ \widehat{\delta^{n-1}\pi} \right]}_{i_{1}i_{2}\ldots i_{n}}&=\textstyle{\frac{1}{3}}\sum_{j_{1},j_{2},\ldots, j_{n}}\omega^{i_{1}\times j_{1}+i_{2}\times j_{2}+\ldots +i_{n}\times j_{n}}\\ &\quad\times\delta_{j_{1}j_{2}}\delta_{j_{1}j_{3}}\ldots\delta_{j_{1}j_{n}}\\ &=\textstyle{\frac{1}{3}}\sum_{j_{1}}\omega^{j_{1}\times (i_{1}+i_{2}+\ldots+i_{n})}\\ &=\textstyle{\frac{1}{3}}\left(1+\omega^{i_{1}+i_{2}+\ldots +i_{n}}+(\omega^{2})^{i_{1}+i_{2}+\ldots +i_{n}}\right). \end{aligned} \end{array} $$

Indexing by ordered-tripartitions, we conclude that

$$\begin{array}{@{}rcl@{}} \begin{aligned} {\left[ \widehat{\delta^{n-1}\pi} \right]}_{\mu}&=\textstyle{\frac{1}{3}}\left(1+\omega^{i_{1}+i_{2}+\ldots +i_{n}}+(\omega^{2})^{i_{1}+i_{2}+\ldots +i_{n}}\right)\\ &=\textstyle{\frac{1}{3}}\left(1+\omega^{|\mu_{1}|+2|\mu_{2}|}+(\omega^{2})^{|\mu_{1}|+2|\mu_{2}|}\right). \end{aligned} \end{array} $$

Now suppose |*μ*_1_|+2|*μ*_2_|=0 (mod 3), then

$$\begin{array}{@{}rcl@{}} \begin{aligned} {\left[ \widehat{\delta^{n-1}\pi} \right]}_{\mu}=\textstyle{\frac{1}{3}}\left(1+1+1\right)=1. \end{aligned} \end{array} $$

If |*μ*_1_|+2|*μ*_2_|=1 (mod 3), then

$$\begin{array}{@{}rcl@{}} \begin{aligned} {\left[ \widehat{\delta^{n-1}\pi} \right]}_{\mu}=\textstyle{\frac{1}{3}}\left(1+\omega+\omega^{2}\right)=0, \end{aligned} \end{array} $$

and if |*μ*_1_|+2|*μ*_2_|=2 (mod 3), then

$$\begin{array}{@{}rcl@{}} \begin{aligned} {\left[ \widehat{\delta^{n-1}\pi} \right]}_{\mu}=\textstyle{\frac{1}{3}}\left(1+\omega^{2}+\omega\right)=0. \end{aligned} \end{array} $$

Thus we have found a basis where all the elements of the initial star-tree tensor are zero *unless* the tripartion *μ* satisfies |*μ*_1_|+2|*μ*_2_|=0 (mod 3). Crucially, this statement also holds for the phylogenetic tensor $\widehat {P}$ because in this basis the rate matrices of this model are diagonal:

$$\begin{array}{@{}rcl@{}} \begin{aligned} {\left[ \widehat{P} \right]}_{\mu}&={\left[ \widehat{P} \right]}_{\mu_{0}\mathord{:} \mu_{1}\mathord{:} \mu_{2}}\\ &=e^{-\lambda}\exp\left(\textstyle{\frac{1}{2}}\sum_{\emptyset \neq e \subseteq {\left[ n-1 \right]}}{\left[ \alpha_{e} K^{(e)}_{1}\,+\,\beta_{e}K^{(e)}_{2} \right]}^{\mu_{0}\mathord{:} \mu_{1}\mathord{:} \mu_{2}}_{\mu_{0}\mathord{:} \mu_{1}\mathord{:} \mu_{2}}\right)\\ &\hspace{6em}\times\textstyle{\frac{1}{3}}\left(1+\omega^{1|\mu_{1}|}+\omega^{2|\mu_{2}|}\right). \end{aligned} \end{array} $$

We deal with this condition on *μ* by taking *u*=*u*_0_*:u*_1_*:u*_2_ as an ordered-tripartion of the reduced set [*n*−1] and setting *μ*=*u*·*γ*(*u*) (considered as the concatenation of strings) where

$$\begin{array}{@{}rcl@{}} \begin{aligned} \gamma(u)&=\left\{ \begin{array}{ll} 0,&\;\;\text{if}\;|u_{1}|+2|u_{2}|=0\\ 1,&\;\;\text{if}\;|u_{1}|+2|u_{2}|=2 \\ 2;&\;\;\text{if}\;|u_{1}|+2|u_{2}|=1 \end{array}\right.\\ &=3-\left(0|u_{0}|+1|u_{1}|+2|u_{2}|\right)\text{(mod 3)}. \end{aligned} \end{array} $$

If we make the definitions

$$\begin{array}{@{}rcl@{}} \begin{aligned} \mathcal{P}_{u}&:={\left[ \widehat{P} \right]}_{u\cdot \gamma(u)},\qquad\\ \eta_{u}&:={\left[ \sum_{\emptyset \neq e\subseteq\left[n-1\right]}\alpha_{e}K_{1}^{(e)}+\beta_{e}K^{(e)}_{2} \right]}^{u\cdot \gamma(u)}_{u\cdot \gamma(u)},\\ \end{aligned} \end{array} $$

we then have the first part of the inversion

(7) $$\begin{array}{@{}rcl@{}} \begin{aligned} \mathcal{P}_{u}=e^{-\lambda}\exp\left(\eta_{u}\right),\qquad \eta_{u}=\ln\left(\mathcal{P}_{u}\right)+\lambda. \end{aligned} \end{array} $$

As in the $\mathbb {Z}_{2}$ case, we would like to use *η*_*u*_ to recover the rate parameters *α*_*e*_,*β*_*e*_ for all *∅*≠*e*⊆[*n*−1] and thus complete the full inversion for this model. Of course, it is little bit more difficult this time.

Recall that *μ*=*μ*_0_*:μ*_1_*:μ*_2_ with *μ*_*i*_⊆[*n*], whereas *u*=*u*_0_*:u*_1_*:u*_2_ with *u*_*i*_⊆[*n*−1], and *∅*≠*e*⊆[*n*−1]. Considering

$$\begin{array}{@{}rcl@{}} \begin{aligned} {\left[ K^{(e)}_{1} \right]}^{\mu}_{\mu}&=\omega^{|e\cap \mu_{1}|+2|e\cap \mu_{2}|},\\ \end{aligned} \end{array} $$

it follows that

$$\begin{array}{@{}rcl@{}} \begin{aligned} {\left[ K^{(e)}_{1} \right]}^{u\cdot \gamma(u)}_{u\cdot \gamma(u)}&=\omega^{|e\cap u_{1}|+2|e\cap u_{2}|}, \end{aligned} \end{array} $$

and similarly

$$\begin{array}{@{}rcl@{}} \begin{aligned} {\left[ K^{(e)}_{2} \right]}^{u\cdot \gamma(u)}_{u\cdot \gamma(u)}=\omega^{|e\cap u_{2}|+2|e\cap u_{1}|}. \end{aligned} \end{array} $$

We make the observation that

$$\begin{array}{@{}rcl@{}} \begin{aligned} {\left[ F_{1} \right]}_{u}^{e}:={\left[ f^{(n-1)} \right]}_{u_{0}\mathord{:} u_{1}\mathord{:} u_{2}}^{{e}^{c}\mathord{:} e\mathord{:} \emptyset}=\omega^{|u_{1}\cap e|+2|u_{2}\cap e|}={\left[ K^{(e)}_{\alpha} \right]}^{u\cdot \gamma(u)}_{u\cdot \gamma(u)}, \end{aligned} \end{array} $$

and

$$\begin{array}{@{}rcl@{}} \begin{aligned} {\left[ F_{2} \right]}_{u}^{e}:={\left[ f^{(n-1)} \right]}_{u_{0}\mathord{:} u_{1}\mathord{:} u_{2}}^{e^{c}\mathord{:} \emptyset\mathord{:} e}=\omega^{|u_{2}\cap e|+2|u_{1}\cap e|}={\left[ K^{(e)}_{\beta} \right]}^{u\cdot \gamma(u)}_{u\cdot \gamma(u)}, \end{aligned} \end{array} $$

where *F*_1_ and *F*_2_ are 2^*n*−1^×3^*n*−1^ matrices.

Thus we may write

$$\begin{array}{@{}rcl@{}}\begin{aligned} \eta_{u}=\sum_{\emptyset \neq e\subseteq {\left[ n-1 \right]}}\alpha_{e} {\left[ F_{1} \right]}_{u}^{e}+\beta_{e}{\left[ F_{2} \right]}_{u}^{e}. \end{aligned}\end{array} $$

Defining the column vectors $\vec {\alpha }=\{\alpha _{e}\},\vec {\beta }=\{\beta _{e}\}$ and $\vec {\eta }=\{\eta _{u}\}$, we can write

$$\begin{array}{@{}rcl@{}} \begin{aligned} \vec{\eta}=F_{1}\vec{\alpha}+F_{2}\vec{\beta}, \end{aligned} \end{array} $$

and define two 3^*n*−1^×2^*n*−1^ matrices *G*_1_ and *G*_2_ as

$$\begin{array}{@{}rcl@{}} \begin{aligned} {\left[ G_{1} \right]}_{e}^{u}:={\left[ {f^{-1}}^{(n-1)} \right]}_{{e}^{c}\mathord{:} e\mathord{:} \emptyset}^{u},\qquad {\left[ G_{2} \right]}_{e}^{u}:={\left[ {f^{-1}}^{(n-1)} \right]}_{e^{c}\mathord{:} \emptyset\mathord{:}e}^{u}, \end{aligned} \end{array} $$

where

$$\begin{array}{@{}rcl@{}} \begin{aligned} f^{-1}=\left(\begin{array}{ccc} 1 & 1 & 1 \\ 1 & \omega & \omega^{2} \\ 1 & \omega^{2} & \omega \\ \end{array} \right), \end{aligned} \end{array} $$

with *ff*^−1^=**1**.

Considering that

$$\begin{array}{@{}rcl@{}} \begin{aligned} \sum_{v}{\left[\, {f^{-1}}^{(n-1)} \right]}_{u}^{v}{\left[\, f^{(n-1)} \right]}_{v}^{w}=\delta_{uw}, \end{aligned} \end{array} $$

for all ordered-triparitions *u*,*w* of [*n*−1], we have the matrix products

$$\begin{array}{@{}rcl@{}} \begin{aligned} \begin{array}{cc} G_{1} F_{1}=\mathbf{1}, & G_{1} F_{2} =0, \\ G_{2} F_{2}=\mathbf{1}, & G_{2} F_{1} =0. \end{array} \end{aligned} \end{array} $$

Thus the second part of the inversion for this model is

$$\begin{array}{@{}rcl@{}} \begin{aligned} \vec{\alpha}=G_{1}\vec{\eta},\qquad \vec{\beta}=G_{2}\vec{\eta}. \end{aligned} \end{array} $$

Together with (7), these equations give a one-one map between pattern probabilities and edge weights for the group-based model with $G=\mathbb {Z}_{3}$.

### Inversion of the K3ST model

We now consider the K3ST model [36] which occurs as the group-based model with

$$\begin{aligned} G&=\mathbb{Z}_{2}\times \mathbb{Z}_{2}=\{(0,0),(0,1),(1,0),(1,1)\}_{+\text{(mod 2)}}\\ &\cong {\langle (12)(34),(13)(24) \rangle}. \end{aligned} $$

In this model a generic rate matrix is given by

$$\begin{array}{@{}rcl@{}} \begin{aligned} Q=-\left(\alpha+\beta+\gamma\right)\mathbf{1}+\alpha K_{01}+\beta K_{10}+\gamma K_{11}, \end{aligned} \end{array} $$

where

(8) $$\begin{array}{@{}rcl@{}} \begin{aligned} K_{01}&=\mathbf{1}\otimes K=\left(\begin{array}{cccc} 0 & 1 & 0 & 0 \\ 1 & 0 & 0 & 0 \\ 0 & 0 & 0 & 1 \\ 0 & 0 & 1 & 0 \end{array}\right),\quad \\ K_{10}&=K\otimes \mathbf{1}=\left(\begin{array}{cccc} 0 & 0 & 1 & 0 \\ 0 & 0 & 0 & 1 \\ 1 & 0 & 0 & 0 \\ 0 & 1 & 0 & 0 \end{array}\right),\\ K_{11}&=K\otimes K=\left(\begin{array}{cccc} 0 & 0 & 0 & 1 \\ 0 & 0 & 1 & 0 \\ 0 & 1 & 0 & 0 \\ 1 & 0 & 0 & 0 \end{array}\right). \end{aligned} \end{array} $$

We already know that the 2×2 Hadamard matrix *h* diagonalizes *K*, so we see immediately that *H*=*h*⊗*h* diagonalizes this model:

$$\begin{array}{@{}rcl@{}} \begin{aligned} \widehat{K}_{01}:&={HK}_{01} H^{-1}=\mathbf{1}\otimes hKh^{-1}= \left(\begin{array}{cccc} 1 & 0 & 0 & 0 \\ 0 & -1 & 0 & 0 \\ 0 & 0 & 1 & 0 \\ 0 & 0 & 0 & -1 \end{array}\right),\\ \widehat{K}_{10}:&={HK}_{10} H^{-1}=\left(\begin{array}{cccc} 1 & 0 & 0 & 0 \\ 0 & 1 & 0 & 0 \\ 0 & 0 & -1 & 0 \\ 0 & 0 & 0 & -1 \end{array}\right),\\ \widehat{K}_{11}:&={HK}_{11} H^{-1}=\left(\begin{array}{cccc} 1 & 0 & 0 & 0 \\ 0 & -1 & 0 & 0 \\ 0 & 0 & -1 & 0 \\ 0 & 0 & 0 & 1 \end{array}\right). \end{aligned} \end{array} $$

Of course *H* is the character table of $\mathbb {Z}_{2}\times \mathbb {Z}_{2}$ and the permutation matrices (8), together with *K*_00_:=**1**, give the regular representation *ρ*_reg_≅*id*⊗*id*⊕*id*⊗*sgn*⊕*sgn*⊗*id*⊕*sgn*⊗*sgn*, where we recall the basic result that the tensor product of two irreducible representations of a group *G* gives an irreducible representation of *G*×*G*.

Simplifying notation, for this model we index tensors with indices given as pairs: $i,j\,=\,00,01,10,11 \!\in \! \mathbb {Z}_{2} \!\times \! \mathbb {Z}_{2}$; and we express the individual parts using lower case Roman characters. For example, we write *i*:=*ab*=01, with *a*=0 and *b*=1. This gives matrix elements:

$$\begin{array}{@{}rcl@{}} \begin{aligned} {\left[ \widehat{K}_{01} \right]}_{ab}^{cd}&=\delta_{ac}\delta_{bd}(-1)^{b},\quad {\left[ \widehat{K}_{10} \right]}_{ab}^{cd}=\delta_{ac}\delta_{bd}(-1)^{a},\\ {\left[ \widehat{K}_{11} \right]}_{ab}^{cd}&=\delta_{ac}\delta_{bd}(-1)^{a+b}; \end{aligned} \end{array} $$

and more complicated tensor products such as

$$\begin{array}{@{}rcl@{}} \begin{aligned} &{\left[ \widehat{K}_{01}\otimes \widehat{K}_{01}\otimes \mathbf{1} \right]}_{a_{1}b_{1}a_{2}b_{2} a_{3}b_{3}}^{c_{1}d_{1}c_{2}d_{2}c_{3}d_{3}}\\ &\qquad\quad=\delta_{a_{1}c_{1}}\delta_{b_{1}d_{1}}\delta_{a_{2}c_{2}} \delta_{b_{2}d_{2}}\delta_{a_{3}c_{3}}\delta_{b_{3}d_{3}}(-1)^{b_{1}+b_{2}}. \end{aligned} \end{array} $$

Again we interpret strings such as *μ*≡*a*_1_*a*_2_…*a*_*n*_ and *ν*≡*b*_1_*b*_2_…*b*_*n*_ as ordered-bipartitions *μ*=*μ*_0_*:μ*_1_ and *ν*=*ν*_0_*:ν*_1_ of the set [*n*]. We can then write matrix elements of tensor products as

$$\begin{array}{@{}rcl@{}} \begin{aligned} {\left[ \widehat{K}^{(e)}_{01} \right]}_{\mu,\nu}^{\mu',\nu'}&=\delta_{\mu\mu'}\delta_{\nu\nu'}(-1)^{|e\cap \nu_{1}|},\qquad\\ {\left[ \widehat{K}^{(e)}_{10} \right]}_{\mu,\nu}^{\mu',\nu'}&=\delta_{\mu\mu'}\delta_{\nu\nu'}(-1)^{|e\cap \mu_{1}|},\\ {\left[ \widehat{K}^{(e)}_{11} \right]}_{\mu,\nu}^{\mu',\nu'}&=\delta_{\mu\mu'}\delta_{\nu\nu'}(-1)^{|e\cap \mu_{1}|+|e\cap \nu_{1}|}. \end{aligned} \end{array} $$

Taking the stationary distribution $\pi =\frac {1}{4}(1,1,1,1)^{T}$ as initial distribution, the zero edge-length star-tree distribution is given by

$$\begin{array}{@{}rcl@{}} \begin{aligned} {\left[ \delta^{n-1}\pi \right]}_{i_{1}i_{2}\ldots i_{n}}=\textstyle{\frac{1}{4}}\delta_{i_{1}i_{2}}\delta_{i_{1}i_{3}}\ldots \delta_{i_{1}i_{n}}, \end{aligned} \end{array} $$

which in the finer index representation is

$$\begin{array}{@{}rcl@{}} \begin{aligned} &{\left[ \delta^{n-1}\pi \right]}_{a_{1}b_{1}a_{2}b_{2}\ldots a_{n}b_{n}}\\ &\qquad\quad=\textstyle{\frac{1}{4}}\delta_{a_{1}a_{2}}\delta_{a_{1}a_{3}}\ldots \delta_{a_{1}a_{n}}\delta_{b_{1}b_{2}}\delta_{b_{1}b_{3}}\ldots \delta_{b_{1}b_{n}}. \end{aligned} \end{array} $$

Recall that elements of the Hadamard matrix can be written as ${\left [ h \right ]}^{a}_{b}=(-1)^{a\times b}$, where $a,b\in \mathbb {Z}_{2}$ and we allow multiplication × by extending to the ring of integers . In the transformed basis, we have

$$\begin{array}{@{}rcl@{}} \begin{aligned} {\left[ \widehat{{\delta^{n-1}\pi}} \right]}_{a_{1}b_{1}a_{2}b_{2}\ldots a_{n}b_{n}}&={\left[ \widehat{\delta^{n-1}\pi} \right]}_{\mu,\nu}\\ &=\textstyle{\frac{1}{4}}\sum_{c_{1},c_{2},\ldots,c_{n}}^{d_{1},d_{2},\ldots,d_{n}}{\left[ h \right]}^{a_{1}}_{c_{1}}{\left[ h \right]}^{a_{2}}_{c_{2}}\ldots {\left[ h \right]}^{a_{n}}_{c_{n}}{\left[ h \right]}^{b_{1}}_{d_{1}}{\left[ h \right]}^{b_{2}}_{d_{2}}\ldots \\ &\times {\left[ h \right]}^{b_{n}}_{d_{n}}\delta_{a_{1}a_{2}}\delta_{a_{1}a_{3}}\ldots \delta_{a_{1}a_{n}}\delta_{b_{1}b_{2}}\delta_{b_{1}b_{3}}\ldots \delta_{b_{1}b_{n}}\\ &=\textstyle{\frac{1}{4}}\sum_{c_{1},d_{1}}(-1)^{(a_{1}+a_{2}+\ldots a_{n})\times c_{1}+(b_{1}+b_{2}+\ldots +b_{n})\times d_{1}}\\ &=\textstyle{\frac{1}{4}}\left(1+(-1)^{a_{1}+\ldots +a_{n}}+(-1)^{b_{1}+\ldots +b_{n}}{\vphantom{\frac{b}{b}}}\right.\\ &\left. +(-1)^{a_{1}+\ldots +a_{n}+b_{1}+\ldots +b_{n}}\right)\\ &=\left\{\begin{array}{ll} 0,&\;\;\text{if either}\;|\mu_{1}|\;\text{or}\;|\nu_{1}|~\text{is odd};\\ 1,&\;\;\text{if}\;|\mu_{1}|\;\text{and}\;|\nu_{1}|\;\text{are both even}.\end{array}\right. \end{aligned} \end{array} $$

We recall (5), so under this model we can express a generic phylogenetic tensor as

$$\begin{array}{@{}rcl@{}} \begin{aligned} P=e^{-\lambda}\exp\!\left(\sum_{\emptyset \neq e\subseteq {\left[ n-1 \right]}}\alpha_{e}K^{(e)}_{01}\,+\,\beta_{e}K^{(e)}_{10}\,+\,\gamma_{e}K^{(e)}_{11}\right) \cdot \delta^{n-1}\pi. \end{aligned} \end{array} $$

To exclude the vanishing components we define, for all ordered bipartitions *u*=*u*_0_*:u*_1_ of the reduced set [*n*−1],

$$\begin{array}{@{}rcl@{}} \begin{aligned} \gamma(u)&=\left\{ \begin{array}{ll} 0,\;\;\text{if}\;|u_{1}|~\text{is even},\\1,\;\;\text{if}\;|u_{1}|~\text{is odd};\end{array}\right.\\ &=2-(0|u_{0}|+1|u_{1}|) \text{(mod 2)}, \end{aligned} \end{array} $$

and intepret *u*·*γ*(*u*) as the string *u*·*γ*(*u*)=*a*_1_*a*_2_…*a*_*n*−1_*γ*(*u*). Then, for each pair *u*,*v* of ordered-bipartitions of [*n*−1], we define

$$\begin{array}{@{}rcl@{}} \begin{aligned} \eta_{u,v}:={\left[ \sum_{\emptyset \neq e\subseteq \left[n-1\right]}\alpha_{e}K^{(e)}_{01}+\beta_{e}K^{(e)}_{10}+\gamma_{e}K^{(e)}_{11} \right]}^{u\cdot \gamma(u),v\cdot \gamma(v)}_{u\cdot \gamma(u),v\cdot \gamma(v)}, \end{aligned} \end{array} $$

and

$$\begin{array}{@{}rcl@{}} \begin{aligned} \mathcal{P}_{u,v}:={\left[ P \right]}_{u\cdot \gamma(u),v\cdot \gamma(v)}, \end{aligned} \end{array} $$

This gives the inversion

$$\begin{array}{@{}rcl@{}} \begin{aligned} \mathcal{P}_{u,v}=e^{-\lambda}\exp\left(\eta_{u,v}\right),\qquad \eta_{u,v}=\lambda+\ln\left(\mathcal{P}_{u,v}\right). \end{aligned} \end{array} $$

Consider the 2^*n*^×2^*n*−1^ rectangular matrices *F*_01_, *F*_10_ and *F*_11_ with components

$$\begin{array}{@{}rcl@{}} \begin{aligned} \begin{array}{ll} {\left[ F_{01} \right]}_{u,v}^{e}={\left[ K^{(e)}_{01} \right]}^{u,v}_{u,v}=(-1)^{|e\cap v_{1}|}, \\ {\left[ F_{10} \right]}_{u,v}^{e}={\left[ K^{(e)}_{10} \right]}^{u,v}_{u,v}=(-1)^{|e\cap u_{1}|},\\ {\left[ F_{11} \right]}_{u,v}^{e}={\left[ K^{(e)}_{11} \right]}^{u,v}_{u,v}=(-1)^{|e\cap u_{1}|+|e\cap v_{1}|}; \end{array} \end{aligned} \end{array} $$

where *e*⊆[*n*−1] and *u*=*u*_0_*:u*_1_ and *v*=*v*_0_*:v*_1_ are ordered-bipartitions of [*n*−1]. If we define the column vector $\vec {\eta }:=\{\eta _{u,v}\}$ indexed by pairs of ordered-bipartitions and the column vectors $\vec {\alpha }:=\{\alpha _{e}\}$, $\vec {\beta }:=\{\alpha _{e}\}$ and $\vec {\gamma }:=\{\alpha _{e}\}$ indexed by subsets of [*n*−1], we then have the matrix equation

$$\begin{array}{@{}rcl@{}} \begin{aligned} \vec{\eta}=F_{01}\vec{\alpha}+F_{10}\vec{\beta}+F_{11}\vec{\gamma}. \end{aligned} \end{array} $$

Writing *H*^(*n*)^=*H*^(*n*−1)^⊗*H* with *H*^(1)^=*H*, we note that

$$\begin{array}{@{}rcl@{}} \begin{aligned} {\left[ F_{01} \right]}_{u,v}^{e}&={\left[ H^{(n-1)} \right]}_{u,v}^{\emptyset,e},\qquad {\left[ F_{10} \right]}_{u,v}^{e}={\left[ H^{(n-1)} \right]}_{u,v}^{e,\emptyset},\\ {\left[ F_{11} \right]}_{u,v}^{e}&={\left[ H^{(n-1)} \right]}_{u,v}^{e,e}; \end{aligned} \end{array} $$

and define the 2^*n*−1^×2^*n*^ rectangular matrices *G*_01_,*G*_10_ and *G*_11_ as

$$\begin{array}{@{}rcl@{}} \begin{aligned} {\left[ G_{01} \right]}_{e}^{u,v}&={\left[ {H^{-1}}^{(n-1)} \right]}_{\emptyset,e}^{u,v},\qquad {\left[ G_{10} \right]}_{e}^{u,v}={\left[ {H^{-1}}^{(n-1)} \right]}_{e,\emptyset}^{u,v},\\ {\left[ G_{11} \right]}_{e}^{u,v}&={\left[ {H^{-1}}^{(n-1)} \right]}_{e,e}^{u,v}. \end{aligned} \end{array} $$

Noting that

$$\begin{array}{@{}rcl@{}} \begin{aligned} \sum_{w,x}{\left[ {H^{-1}}^{(n-1)} \right]}_{u,v}^{w,x}{\left[ H^{(n-1)} \right]}_{w,x}^{y,z}=\delta_{u,y}\delta_{v,z}, \end{aligned} \end{array} $$

for all *u*,*v*,*y*,*z* ordered-bipartitions of [*n*−1], we then have the matrix identities

$$\begin{array}{@{}rcl@{}} \begin{aligned} G_{01} F_{01}=\mathbf{1},\qquad G_{10} F_{10}=\mathbf{1}, \qquad G_{11} F_{11}=\mathbf{1}, \end{aligned} \end{array} $$

and

$$\begin{array}{@{}rcl@{}} \begin{aligned} G_{01}F_{10}&=0=G_{01} F_{11}=G_{\beta} F_{11}=G_{10} F_{01}\\ &=G_{11} F_{01}=G_{11} F_{10}. \end{aligned} \end{array} $$

Writing

$$\begin{array}{@{}rcl@{}} \begin{aligned} \vec{\alpha}=G_{01} \vec{\eta},\qquad \vec{\beta}=G_{10}\vec{\eta}, \qquad \vec{\gamma}=G_{11}\vec{\eta}, \end{aligned} \end{array} $$

completes the inversion for the K3ST model.

### Inversion of the $\mathbb {Z}_{r}$ model

We now consider the group based model for $\mathbb {Z}_{r}=\left \{0,1,2,\ldots (r-1)\right \}_{+(\text {mod r})}\cong {\langle \sigma :\sigma ^{r}=e \rangle }$. For this model the generic rate matrix has the form

$$\begin{array}{@{}rcl@{}} \begin{aligned} Q=-\lambda\mathbf{1} +\sum_{i=1}^{r}\alpha^{i}K_{\sigma^{i}}, \end{aligned} \end{array} $$

where $\lambda =\sum _{i=1}^{r}\alpha ^{i}$ and

$$\begin{array}{@{}rcl@{}} \begin{aligned} K_{\sigma}=\left(\begin{array}{ccccc} 0 & 0 & \ldots & 0 & 1 \\ 1 & 0 & 0 & 0 & 0 \\ 0 & 1 & \ldots & 0 & 0 \\ \vdots & \vdots & \ddots & \vdots & \vdots \\ 0 & 0 & \ldots & 1 & 0 \end{array}\right), \end{aligned} \end{array} $$

so that $K_{\sigma ^{i}}=K_{\sigma }^{i}$.

Defining *ω*=*e*^2*πi*/*r*^, we have *ω*^*r*^=1 and 1+*ω*+*ω*^2^+…+*ω*^*r*−1^=0 and ${\left [ f \right ]}_{i}^{j}=\omega ^{ij}$ where *i*,*j*=0,1,2,…,*r*−1. Of course, *f* is the character table of $\mathbb {Z}_{r}$ and ${\left [\, f^{-1} \right ]}^{i}_{j}=\textstyle {\frac {1}{r}}\omega ^{-ij}$.

#### **Lemma1**.

$$\begin{array}{@{}rcl@{}} \begin{aligned} \sum_{\nu}{\left[\, f\otimes f\otimes \ldots \otimes f \right]}_{\mu}^{\nu}{\left[\, f^{-1}\otimes f^{-1}\otimes \ldots \otimes f^{-1} \right]}_{\nu}^{\mu'}=\delta_{\mu\mu'}, \end{aligned} \end{array} $$

where *μ*,*ν*,*μ*^′^ are ordered-*r*-partitions of the set [*n*] defined by the strings *i*_1_*i*_2_…*i*_*n*_, *j*_1_*j*_2_…*j*_*n*_ and *k*_1_*k*_2_…*k*_*n*_, respectively.

#### *Proof*.

The result is obvious by the definition of tensor product. However, explicitly we have

$$\begin{array}{@{}rcl@{}}\begin{aligned} \sum_{\nu}{\left[\, f\otimes f\otimes \ldots \otimes f \right]}_{\mu}^{\nu}&{\left[\, f^{-1}\otimes f^{-1}\otimes \ldots \otimes f^{-1} \right]}_{\nu}^{\mu'}\\ &=\textstyle{\frac{1}{r^{n}}}\sum_{0\leq j_{1},j_{2},\ldots,j_{r-1}\leq (r-1)}\\ &\times\omega^{i_{1}j_{1}+i_{2}j_{2}+\ldots i_{r-1}j_{r-1}}\omega^{-\left(j_{1}k_{1}+j_{2}k_{2}+\ldots +j_{n}k_{n}\right)}\\ &=\textstyle{\frac{1}{r^{n}}}\sum_{0\leq j_{1},j_{2},\ldots,j_{r-1}\leq (r-1)}\\ &\times\omega^{\,j_{1}(i_{1}-k_{1})+j_{2}(i_{2}-k_{2})+\ldots +j_{n}(i_{n}-k_{n})} \end{aligned} \end{array} $$

which clearly equals 1 if *i*_*ℓ*_−*k*_*ℓ*_=0 for all *ℓ*, and, by repeatedly applying 1+*ω*+*ω*^2^+…+*ω*^*r*−1^=0, equals 0 otherwise.

The regular representation contains exactly one copy of every irreducible representation and the irreducible representations of $\mathbb {Z}_{r}$ are given by the powers of *ω*:

$$\rho_{i}: \begin{array}{cc}\mathbb{Z}_{r}\rightarrow \mathbb{C} \\ \sigma\mapsto \omega^{i} \end{array}. $$

Thus the change of basis $K_{\sigma ^{i}}\mapsto \widehat {K}_{\sigma ^{i}}={fK}_{\sigma ^{i}}f^{-1}$ will give diagonal matrices $\widehat {K}_{\sigma ^{i}}$. Additionally,

#### **Lemma2**.

In the diagonal basis, the matrices $\widehat {K}_{\sigma ^{i}}:={fK}_{\sigma ^{i}}f^{-1}$ have matrix elements given by ${\left [ \widehat {K}_{\sigma ^{s}} \right ]}_{i}^{j}=\omega ^{is}\delta _{\textit {ij}}$.

#### *Proof*.

Consider the matrix elements ${\left [ K_{\sigma ^{s}} \right ]}_{i}^{j}=\delta _{i\sigma ^{s}(j)}$. Thus

$$\begin{array}{@{}rcl@{}} \begin{aligned} {\left[\, {fK}_{\sigma^{s}}f^{-1} \right]}^{i}_{j}&=\sum_{k,l}\omega^{ik}\delta_{k\sigma^{s}(l)}\omega^{-lj}=\sum_{l}\omega^{i\sigma^{s}(l)-lj}\\ &=\sum_{l}\omega^{i(l+s)-lj}\\ &=\omega^{is}\sum_{l}\omega^{l(i-j)}=\omega^{is}\delta_{ij}, \end{aligned} \end{array} $$

where we have used $\omega ^{\sigma ^{s}(m)}=\omega ^{m+s}$.

Now

$$\begin{array}{@{}rcl@{}} \begin{aligned} {\left[ \delta^{n-1}\pi \right]}_{i_{1}i_{2}\ldots i_{n}}=\textstyle{\frac{1}{r}}\delta_{i_{1}i_{2}}\delta_{i_{1}i_{3}}\ldots \delta_{i_{1}i_{n}}, \end{aligned} \end{array} $$

and

$$\begin{array}{@{}rcl@{}} \begin{aligned} {\left[ \widehat{\delta^{n-1}\pi} \right]}_{i_{1}i_{2}\ldots i_{n}}&=\textstyle{\frac{1}{r}}\sum_{j_{1},j_{2},\ldots,j_{r}}\omega^{i_{1}j_{1}+i_{2}j_{2}+\ldots +i_{n}j_{n}}\\ &\quad\;\times\delta_{j_{1}j_{2}}\delta_{j_{1}j_{3}}\ldots \delta_{j_{1}j_{n}}\\ &=\textstyle{\frac{1}{r}}\sum_{j_{1}}\omega^{\,j_{1}(i_{1}+i_{2}+\ldots +i_{n})}\\ &=\left\{\begin{array}{ll} 1&\;\;\text{if}\;i_{1}+i_{2}+\ldots +i_{n}=0\text{(mod r)} \\ 0,&\;\;\text{otherwise.} \end{array}\right. \end{aligned} \end{array} $$

Translating this result using the ordered-*r*-partitions for indices, we have

#### **Lemma3**.

In the diagonal basis, the uniform initial distribution on the star tree has components

$$\begin{array}{@{}rcl@{}} {\small\begin{aligned} &{\left[ \widehat{\delta^{n-1}\pi} \right]}_{\mu}\\ &=\left\{\!\begin{array}{ll} {1~~\text{if}\;0|\mu_{0}|\,+\,1|\mu_{1}|\,+\,2|\mu_{2}|\,+\,\ldots \,+\,(r-1)|\mu_{r\,-\,1}|=0 \textit{(mod r)}} \\ {0,~\text{otherwise.}}\end{array}\right.\!, \end{aligned}} \end{array} $$

where *μ*=*μ*_0_*:μ*_1_*:μ*_2_*:*…*:μ*_*r*−1_ is an ordered-*r*-partition of the set [*n*].

Again recall that for this model a generic phylogenetic tensor can be written as

$$\begin{array}{@{}rcl@{}} \begin{aligned} P=e^{-\lambda}\exp\left(\sum_{\emptyset \neq e\subseteq {\left[ n-1 \right]},s\in{\left[ r-1 \right]}} {\alpha_{e}^{s}} K^{(e)}_{\sigma^{s}}\right)\delta^{n-1}\pi, \end{aligned} \end{array} $$

where $\pi =\textstyle {\frac {1}{r}}(1,1,\ldots,1)^{T}$. In the diagonal basis $\widehat {P}:=f^{(n)}\cdot P $ and as a consequence of Lemma 3$\widehat {P}$ will have many vanishing components. To avoid these we take *u*=*u*_0_*:u*_1_*:u*_2_*:*…*:u*_*r*−1_ as an *ordered-r-partition* of [*n*−1] and set

$$\begin{array}{@{}rcl@{}} \begin{aligned} \gamma(u)&=r-\left(0|u_{0}|+1|u_{1}|+2|u_{2}|+\ldots \right.\\ &\quad\, \left.+(r-1)|u_{r-1}|\right) \;\text{(mod r)}. \end{aligned} \end{array} $$

If we define $\mathcal {P}_{u}:={\left [ \widehat {P} \right ]}_{u\cdot \gamma (u)}$ and

$$\begin{array}{@{}rcl@{}} \begin{aligned} \eta_{u}:={\left[\sum_{\emptyset \neq e\subseteq \left[n-1\right],s\in\left[r-1\right]} {\alpha_{e}^{s}} \widehat{K}^{(e)}_{\sigma^{s}} \right]}_{u\cdot \gamma(u)}^{u\cdot \gamma(u)}, \end{aligned} \end{array} $$

we then have the first part of the inversion for the $\mathbb {Z}_{r}$ model:

$$\begin{array}{@{}rcl@{}} \begin{aligned} \mathcal{P}_{u}&=e^{-\lambda}\exp\left(\eta_{u}\right),\\ \eta_{u}&=\ln\left(\mathcal{P}_{u}\right)+\lambda. \end{aligned} \end{array} $$

For each *i*∈[*r*−1], we define the column vectors $\vec {\alpha }_{i} :=\left \{{\alpha _{e}^{i}}\right \}_{\emptyset \neq e\subseteq {\left [ n-1 \right ]}}$, and, for each *∅*≠*e*⊆[*n*−1] and *u* an ordered- (*r*−1)-partition of [*n*−1], we define the rectangular *r*^*n*−1^×2^*n*−1^ matrices

$$\begin{array}{@{}rcl@{}}\begin{aligned} \begin{array}{llll} \;\;\;{\left[ F_{1} \right]}_{u}^{e}&:={\left[ K^{(e)}_{\sigma} \right]}_{u\cdot \gamma(u)}^{u\cdot \gamma(u)}, & {\left[ F_{2} \right]}^{e}_{u}:={\left[ K^{(e)}_{\sigma^{2}} \right]}^{u\cdot \gamma(u)}_{u\cdot \gamma(u)}, \ldots \\ {\left[ F_{r-1} \right]}_{u}^{e}&:={\left[ K^{(e)}_{\sigma^{r-1}} \right]}^{u\cdot \gamma(u)}_{u\cdot \gamma(u)}, \end{array} \end{aligned} \end{array} $$

so we have the vector equation

$$\begin{array}{@{}rcl@{}} \begin{aligned} \eta=F_{1}\vec{\alpha_{1}}+F_{2}\vec{\alpha_{2}}+\ldots + F_{r-1}\vec{\alpha}_{r-1}. \end{aligned} \end{array} $$

We claim that

#### **Lemma4**.

$$\begin{array}{@{}rcl@{}} \begin{aligned} {\left[ F_{1} \right]}^{e}_{u}&={\left[\, f^{(n-1)} \right]}^{e^{c}\mathord{:} e\mathord{:} \emptyset\mathord{:} \emptyset\mathord{:} \ldots\mathord{:} \emptyset}_{u}, \qquad\\ {\left[ F_{2} \right]}^{e}_{u}&={\left[\, f^{(n-1)} \right]}^{e^{c} \mathord{:} \emptyset\mathord{:} e\mathord{:} \emptyset\mathord{:} \ldots\mathord{:} \emptyset}_{u},\quad \cdots \\ \cdots \quad {\left[ F_{r-1} \right]}^{e}_{u}&={\left[\, f^{(n-1)} \right]}^{e^{c}\mathord{:} \emptyset\mathord{:} \emptyset\mathord{:} \emptyset\mathord{:} \ldots\mathord{:} e}_{u}. \end{aligned}\end{array} $$

#### *Proof*.

We recall that ${\left [ \widehat {K}_{\sigma ^{s}} \right ]}_{i}^{j}=\omega ^{is}\delta _{\textit {ij}}$, so, for *μ*=*μ*_0_*:μ*_1_*:μ*_2_*:*…*:μ*_*r*−1_ an ordered-*r*-parition of [*n*], and *e* a subset of [*n*−1] we have

$$\begin{array}{@{}rcl@{}} \begin{aligned} {\left[ \widehat{K}^{(e)}_{\sigma^{s}} \right]}^{\mu}_{\mu}=\omega^{s\left(0|\mu_{0}\cap e|+1|\mu_{1}\cap e|+\ldots +(r-1)|\mu_{r-1}\cap e|\right)}, \end{aligned} \end{array} $$

so

$$\begin{array}{@{}rcl@{}} \begin{aligned} {\left[ \widehat{K}^{(e)}_{\sigma^{s}} \right]}^{u\cdot \gamma(u)}_{u\cdot \gamma(u)}=\omega^{s\left(0|u_{0}\cap e|+1|u_{1}\cap e|+\ldots +(r-1)|u_{r-1}\cap e|\right)}, \end{aligned} \end{array} $$

because *e*⊆[*n*−1]. On the other hand ${\left [\, f \right ]}_{i}^{j}=\omega ^{ij}$, so

$$\begin{array}{@{}rcl@{}}\begin{aligned} {\left[\, f^{(n-1)} \right]}_{u}^{e^{c}\mathord{:} \emptyset\mathord{:} \ldots \mathord{:} \emptyset\mathord{:} e\mathord{:} \emptyset \mathord{:} \ldots \mathord{:} \emptyset}=\omega^{s\left(0|u_{0}\cap e|+1|u_{1}\cap e|+\ldots +(r-1)|u_{r-1}\cap e|\right)}, \end{aligned} \end{array} $$

where *e* appears in the *s*^*th*^ position.

Define, for *i*∈[*r*−1], the rectangular 2^*n*−1^×*r*^*n*−1^ matrices

$$\begin{array}{@{}rcl@{}}\begin{aligned} {\left[ G_{1} \right]}^{u}_{e}:&={\left[\, {f^{-1}}^{(n-1)} \right]}^{e^{c}\cdot \gamma(u)\mathord{:} e\mathord{:} \emptyset\mathord{:} \emptyset\mathord{:} \ldots\mathord{:} \emptyset}_{u\cdot \gamma(u)}\\ {\left[ G_{2} \right]}^{u}_{e}:&={\left[\, {f^{-1}}^{(n-1)} \right]}^{e^{c}\cdot \gamma(u)\mathord{:} \emptyset\mathord{:} e\mathord{:} \emptyset\mathord{:} \ldots\mathord{:} \emptyset}_{u\cdot \gamma(u)}\\ &\vdots\\ {\left[ G_{r-1} \right]}^{u}_{e}&={\left[\, {f^{-1}}^{(n-1)} \right]}^{e^{c}\cdot\gamma(u)\mathord{:} \emptyset\mathord{:} \emptyset\mathord{:} \emptyset\mathord{:} \ldots\mathord{:} e}_{u\cdot \gamma(u)}. \end{aligned} \end{array} $$

Of course *G*_*i*_*F*_*j*_=*δ*_*ij*_**1**, so we now have the second part of the inversion:

$$\begin{array}{@{}rcl@{}} \begin{aligned} \vec{\alpha_{i}}=G_{i}\eta. \end{aligned} \end{array} $$

### Inversion of any abelian group-based model

#### **Lemma5**.

Any (finitely generated) abelian group *G* is isomorphic to a direct product of cyclic groups of prime-power order, ie. $G\cong \mathbb {Z}_{r_{1}}\times \mathbb {Z}_{r_{2}}\times \ldots \times \mathbb {Z}_{r_{q}}$ where each $r_{i}=p_{i}^{n_{i}}$ where *p*_*i*_ is prime and *n*_*i*_ is a positive integer.

#### **Lemma6**.

The group-based model arising from the *G* is defined only up to group isomorphisms of *G*.

#### *Proof*.

A generic rate matrix for the group-based model arsing from *G* is given by

$$\begin{array}{@{}rcl@{}} \begin{aligned} Q=-\lambda\mathbf{1}+\sum_{e\neq \sigma\in G}\alpha^{\sigma}K_{\sigma}. \end{aligned} \end{array} $$

Under a group isomorphism *ϕ*:*G*→*G*^′^, we have *ϕ*(*σ*_*i*_*σ*_*j*_)=*ϕ*(*σ*_*i*_)*ϕ*(*σ*_*j*_).

Recall (2), so that the matrix elements ${\left [ K_{\sigma } \right ]}_{i}^{j}$ is set via the action *σ*_*i*_↦*σσ*_*i*_=*σ*_*j*_. If we consider the regular representation of *G*^′^ we then have ${\left [ K_{\phi (\sigma }) \right ]}_{i}^{j}$ defined by *ϕ*(*σ*_*i*_)↦*ϕ*(*σ*)*ϕ*(*σ*_*i*_). Now *ϕ*(*σ*)*ϕ*(*σ*_*i*_)=*ϕ*(*σσ*_*i*_)=*ϕ*(*σ*_*j*_) and, because *ϕ* is a group isomorphism, this occurs if and only if *σσ*_*i*_=*σ*_*j*_. Thus ${\left [ K_{\phi (\sigma }) \right ]}_{i}^{j}={\left [ K_{\sigma } \right ]}_{i}^{j}$ for all *i* and *j*.

This means that we can restrict attention to a single representitive in the isomorphism class of *G*. Of course, for this purpose we choose the representative guaranteed by Lemma 5.

Thus, for any abelian group *G*, with generators *σ*_1_,*σ*_2_,…,*σ*_*q*_ the corresponding group-based model has rate generators given by

$$\begin{array}{@{}rcl@{}} \begin{aligned} L_{\sigma}=-\mathbf{1}+{K}_{\sigma_{1}^{m_{1}}}\otimes {K}_{\sigma_{2}^{m_{2}}}\otimes \ldots \otimes {K}_{\sigma_{q}^{m_{q}}}, \end{aligned} \end{array} $$

for all $e\neq \sigma =\left (\sigma _{1}^{m_{1}},\sigma _{2}^{m_{2}},\ldots,\sigma _{q}^{m_{q}}\right)\in G$, where $K_{\sigma _{i}}$ is the permutation matrix representing the generator $\sigma _{i}\in \mathbb {Z}_{r_{i}}$. The character table *f* of *G* is simply the tensor product of the individual character tables of the $\mathbb {Z}_{r_{i}}$:

$$\begin{array}{@{}rcl@{}} \begin{aligned} f=f_{1}\otimes f_{2}\otimes \ldots \otimes f_{q}. \end{aligned} \end{array} $$

In the diagonal basis we have matrix elements

$$\begin{array}{@{}rcl@{}} \begin{aligned} {\left[\, f_{k}K_{{\sigma_{k}^{s}}}f_{k}^{-1} \right]}_{i}^{j}={\left[ \hat{K}_{{\sigma_{k}^{s}}} \right]}_{i}^{j}=\left(\omega_{k}\right)^{is}\delta_{ij}, \end{aligned} \end{array} $$

where *ω*_*k*_ is a *k*^*th*^ root of unity. Thus

$$\begin{array}{@{}rcl@{}} \begin{aligned} &{\left[ \hat{K}_{\sigma_{1}^{m_{1}}}\otimes \hat{K}_{\sigma_{2}^{m_{2}}} \otimes \ldots \hat{K}_{\sigma_{q}^{m_{q}}} \right]}_{i_{1}i_{2}\ldots i_{q}}^{j_{1}j_{2}\ldots j_{q}}\\ &\qquad\quad=\delta_{i_{1}j_{1}}\delta_{i_{2}j_{2}}\ldots \delta_{i_{q}j_{q}}\left(\omega_{1}\right)^{i_{1}m_{1}}\left(\omega_{2}\right)^{i_{2}m_{2}}\ldots \left(\omega_{q}\right)^{i_{q}m_{q}}. \end{aligned} \end{array} $$

We write phylogenetic tensors for this model in the form

$$P_{i_{11}i_{12}\ldots i_{1n},i_{21}i_{22}\ldots i_{2n}\ldots \ldots i_{q1}i_{q2}\ldots i_{qn}}, $$

where 0≤*i*_*sj*_≤*r*_*s*_ for all 0≤*s*≤*q*. We simplify notation by writing each group of indices as *μ*^(*s*)^:=*i*_*s*1_*i*_*s*2_…*i*_*sn*_ where *μ*^(*s*)^ is an ordered- *r*_*s*_-partition of [*n*].

#### **Lemma7**.

In the diagonal basis, the uniform initial distribution on the star tree has components

$$\begin{array}{@{}rcl@{}}\begin{aligned} &{\left[ \widehat{\delta^{n-1}\pi} \right]}_{\mu^{(1)}\mu^{(2)}\ldots \mu^{(q)}}\\ &\quad=\left\{\begin{array}{ll}1,&\;\;\text{if}\;0|\mu_{0}^{(i)}|+\!1|\mu_{1}^{(i)}|+\ldots +(r_{i}\,-\,1)|\mu_{r-1}^{(i)}|=0\forall i;\\ 0,&\;\;\text{otherwise.}\end{array}\right. \end{aligned}\end{array} $$

A generic phylogenetic tensor for this model can be expressed as

$$\begin{array}{@{}rcl@{}} {\small\begin{aligned} P=e^{-\lambda}\!\exp\!\left(\sum_{\emptyset \neq e\subseteq {\left[ n-1 \right]}}^{s_{i}\in{\left[ r_{i}-1 \right]}}\!\!\alpha^{s_{1}s_{2}\ldots s_{q}}_{e}K^{(e)}_{\sigma^{s_{1}}_{1}}\!\otimes\! K^{(e)}_{\sigma^{s_{2}}_{2}}\!\otimes\! \ldots \!\otimes\! K^{(e)}_{\sigma^{s_{q}}_{q}}\!\right)\!\cdot \delta^{n\,-\,1}\pi,  \end{aligned}} \end{array} $$

where *π* is the unifrom distribution on ${\sum _{i=1}^{q} r_{i}}$ states, i.e.

$$\pi=\left({\sum_{i=1}^{q} r_{i}}\right)^{-1}\left(1,1,\ldots,1\right)^{T}. $$

In the diagonal basis $\widehat {P}=(\,f_{1}\otimes f_{2}\otimes \ldots \otimes f_{q})^{(n)}\cdot P$, and, as a consequence of the previous lemma, *P* has many vanishing components. To avoid these, for each *i*∈[*q*] we take $u^{(i)}=u^{(i)}_{0}\mathord {:} u^{(i)}_{1}\mathord {:} u^{(i)}_{2}\mathord {:} \ldots \mathord {:} u^{(i)}_{r_{i}-1} $ as an *ordered- r*_*i*_*-partition* of [*n*−1] and set

$$\begin{array}{@{}rcl@{}} \begin{aligned} \gamma_{i}(u^{(i)})&=r_{i}-\!\left(0|u^{(i)}_{0}|+1|u^{(i)}_{1}|+2|u^{(i)}_{2}|+\ldots \right. \\ &\qquad\qquad \left.+(r_{i}-1)|u^{(i)}_{r-1}|\right) (\text{mod} r). \end{aligned} \end{array} $$

We then define

$$\begin{array}{@{}rcl@{}} \begin{aligned} \mathcal{P}_{u^{(1)}u^{(2)}\ldots u^{(q)}}:={\left[ \widehat{P} \right]}_{u^{(1)}\cdot \gamma_{1}\left(u^{(1)}\right)u^{(2)}\cdot \gamma_{2}\left(u^{(2)}\right)\ldots u^{(q)}\cdot \gamma_{1}\left(u^{(q)}\right)}, \end{aligned} \end{array} $$

and

$$\begin{array}{@{}rcl@{}} {\small\begin{aligned} &\eta_{u^{(1)}\ldots u^{(q)}}\\ &:=\!{\left[ \sum_{\emptyset \neq e\subseteq \left[n-1\right]}^{s_{i}\in\left[r_{i}-1\right]} \!\alpha^{s_{1}\ldots s_{q}}_{e}\widehat{K}^{(e)}_{\sigma^{s_{1}}_{1}}\otimes \ldots \otimes \widehat{K}^{(e)}_{\sigma^{s_{q}}_{q}} \right]}^{u^{(1)}\cdot \gamma_{1}\left(u^{(1)}\right)\ldots u^{(q)}\cdot \gamma_{1}\left(u^{(q)}\right)}_{u^{(1)}\cdot \gamma_{1}\left(u^{(1)}\right)\ldots u^{(q)}\cdot \gamma_{1}(u^{(q)})}, \end{aligned}} \end{array} $$

so that we have the first part of the inversion

$$\begin{array}{@{}rcl@{}}\begin{aligned} \mathcal{P}_{u^{(1)}u^{(2)}\ldots u^{(q)}}&=e^{-\lambda}\exp\left(\eta_{u^{(1)}u^{(2)}\ldots u^{(q)}}\right),\\ \eta_{u^{(1)}u^{(2)}\ldots u^{(q)}}&=\lambda+\ln\left(\mathcal{P}_{u^{(1)}u^{(2)}\ldots u^{(q)}}\right).  \end{aligned} \end{array} $$

We define the column vectors $\vec {\alpha }^{s_{1}s_{2}\ldots s_{q}}:= \left \{\alpha ^{s_{1}s_{2}\ldots s_{q}}_{e}\right \}_{\emptyset \neq e\subseteq {\left [ n-1 \right ]}}\vspace *{2pt}$ and $\vec {\eta }:=\{\eta _{u^{(1)}u^{(2)}\ldots u^{(q)}}\}$ where *u*_*i*_ is an ordered- *r*_*i*_-partition of [*n*−1], and we define the (*r*_1_*r*_2_…*r*_*q*_)^*n*−1^×2^*n*−1^ matrices

$$\begin{array}{@{}rcl@{}}\begin{aligned} &{\left[ F_{s_{1}s_{2}\ldots s_{q}} \right]}^{e}_{u_{1}u_{2}\ldots u_{q}}\\ &\quad:={\left[ K_{\sigma^{s_{1}}_{1}}^{(e)} \right]}^{u_{1}\cdot \gamma(u_{1})}_{u_{1}\cdot \gamma(u_{1})}{\left[ K_{\sigma^{s_{2}}_{2}}^{(e)} \right]}^{u_{2}\cdot \gamma(u_{2})}_{u_{2}\cdot \gamma(u_{2})}\ldots{\left[ K_{\sigma^{s_{q}}_{q}}^{(e)} \right]}^{u_{q}\cdot \gamma(u_{q})}_{u_{q}\cdot \gamma(u_{q})}\\ &\quad\,={\left[\, f_{1}^{(n-1)} \right]}_{u_{1}}^{e^{c}\mathord{:} \emptyset \mathord{:} \ldots \mathord{:} \emptyset \mathord{:} e\mathord{:} \emptyset \mathord{:} \ldots \mathord{:}\emptyset} {\left[\, f_{2}^{(n-1)} \right]}_{u_{2}}^{e^{c}\mathord{:} \emptyset \mathord{:} \ldots \mathord{:} \emptyset \mathord{:} e\mathord{:} \emptyset \mathord{:} \ldots \mathord{:}\emptyset} \ldots\\ &\hspace{13em}\ldots {\left[\, f_{q}^{(n-1)} \right]}_{u_{q}}^{e^{c}\mathord{:} \emptyset \mathord{:} \ldots \mathord{:} \emptyset \mathord{:} e\mathord{:} \emptyset \mathord{:} \ldots \mathord{:}\emptyset},  \end{aligned} \end{array} $$

where in each term *e* appears in the $s_{i}^{th}$ position and the equality follows from Lemma 4.

We can then write the vector equation

$$\begin{array}{@{}rcl@{}} \begin{aligned} \vec{\eta}=\sum_{s_{1}s_{2}\ldots s_{q}:1\leq s_{i}\leq r_{i}-1}F_{s_{1}s_{2}\ldots s_{q}}\vec{\alpha}^{s_{1}s_{2}\ldots s_{q}}. \end{aligned} \end{array} $$

If we define the 2^*n*−1^×(*r*_1_*r*_2_…*r*_*q*_)^*n*−1^ matrices

$$\begin{array}{@{}rcl@{}} \begin{aligned} &{\left[ G_{s_{1}s_{2}\ldots s_{q}} \right]}_{e}^{u_{1}u_{2}\ldots u_{q}}\\ &\quad={\left[\, {f_{1}^{-1}}^{(n-1)} \right]}_{u_{1}}^{e^{c}\mathord{:} \emptyset \mathord{:} \ldots \mathord{:} \emptyset \mathord{:} e\mathord{:} \emptyset \mathord{:} \ldots \mathord{:}\emptyset} &\!\!\!{\left[\, {f_{2}^{-1}}^{(n-1)} \right]}_{u_{2}}^{e^{c}\mathord{:} \emptyset \mathord{:} \ldots \mathord{:} \emptyset \mathord{:} e\mathord{:} \emptyset \mathord{:} \ldots \mathord{:}\emptyset} \ldots\\ &&\!\!\!\ldots {\left[\, {f_{q}^{-1}}^{(n-1)} \right]}_{u_{q}}^{e^{c}\mathord{:} \emptyset \mathord{:} \ldots \mathord{:} \emptyset \mathord{:} e\mathord{:} \emptyset \mathord{:} \ldots \mathord{:}\emptyset},  \end{aligned} \end{array} $$

where in each term *e* appears in the $s_{i}^{th}$ position, we have the orthogonality relations

$$\begin{array}{@{}rcl@{}} \begin{aligned} G_{s_{1}s_{2}\ldots s_{q}}F_{s_{1}'s_{2}'\ldots s_{q}'}=\delta_{s_{1}s_{1}'}\delta_{s_{2}s_{2}'}\ldots \delta_{s_{q}s_{q}'}\mathbf{1}. \end{aligned} \end{array} $$

This gives us the second part of the inversion of any group-based model:

$$\begin{array}{@{}rcl@{}} \begin{aligned} \vec{\alpha}^{s_{1}s_{2}\ldots s_{q}}=G_{s_{1}s_{2}\ldots s_{q}}\vec{\eta}. \end{aligned} \end{array} $$

## Conclusion

In this article we have given an alternative derivation of the inversion of group-based phylogenetic models. Primarily our method relies on the remarkable intertwining relation between branching events and Markov evolution (4), and the resulting simplified expression of phylogenetic tensors given in (5). From there we took a representation theoretic approach concentrating on the structure of tensor indices.
